# Embedding laser generated nanocrystals in BiVO_4_ photoanode for efficient photoelectrochemical water splitting

**DOI:** 10.1038/s41467-019-10543-z

**Published:** 2019-06-13

**Authors:** Jie Jian, Youxun Xu, Xiaokun Yang, Wei Liu, Maosen Fu, Huiwu Yu, Fei Xu, Fan Feng, Lichao Jia, Dennis Friedrich, Roel van de Krol, Hongqiang Wang

**Affiliations:** 1 State Key Laboratory of Solidification Processing, Center for Nano Energy Materials, School of Materials Science and Engineering, Northwestern Polytechnical University and Shaanxi Joint Labortary of Graphene, Xi’an, 710072 P. R. China; 2 Key Laboratory of Applied Surface and Colloid Chemistry, National Ministry of Education, Shaanxi Key Laboratory for Advanced Energy Devices, Shaanxi Engineering Lab for Advanced Energy Technology, School of Materials Science and Engineering, Shaanxi Normal University, 620 West Chang’an Street, Xi’an, 710119 P. R. China; 3 Institute for Solar Fuels, Helmholtz-Zentrum Berlin für Materialien und Energie GmbH, Hahn-Meitner-Platz 1, 14109 Berlin, Germany

**Keywords:** Solid-state chemistry, Energy science and technology, Photocatalysis, Synthesis and processing

## Abstract

Addressing the intrinsic charge transport limitation of metal oxides has been of significance for pursuing viable PEC water splitting photoelectrodes. Growing a photoelectrode with conductive nanoobjects embedded in the matrix is promising for enhanced charge transport but remains a challenge technically. We herein show a strategy of embedding laser generated nanocrystals in BiVO_4_ photoanode matrix, which achieves photocurrent densities of up to 5.15 mA cm^−2^ at 1.23 V_RHE_ (from original 4.01 mA cm^−2^) for a single photoanode configuration, and 6.22 mA cm^−2^ at 1.23 V_RHE_ for a dual configuration. The enhanced performance by such embedding is found universal owing to the typical features of laser synthesis and processing of colloids (LSPC) for producing ligand free nanocrystals in desired solvents. This study provides an alternative to address the slow bulk charge transport that bothers most metal oxides, and thus is significant for boosting their PEC water splitting performance.

## Abstract (web summary)

While photoelectrochemical water splitting offers a low-cost, integrated means to generate fuel from light, poor charge carrier transport limits performances. Here, authors embed laser-synthesized colloids in bismuth vanadate photoanodes to boost charge carrier mobilities and enhance photocurrents.

## Introduction

Photoelectrochemical (PEC) water splitting has been considered as one of the most promising technologies for generating hydrogen using sunlight. Since its discovery in 1970s, searching for robust and efficient photoanode materials has been one of the toughest challenges. III–V semiconductors have demonstrated solar to hydrogen (STH) conversion efficiency over 20% while suffering from severe instability in aqueous solutions as well as cost issues that are incompatible for its commercialization^1,2^. Metal oxides are relatively cheap and show much better stabilities than semiconductors like III–V and II–VI^3^, however, they have so far only demonstrated moderate STH conversion efficiencies (<8%)^4–6^. One of the major limitations of metal oxides is their poor charge carrier transport, which is usually several orders lower than those of III–V or elemental semiconductors, resulting in low performance that are far below their theoretical values^7–10^. Bismuth vanadate (BiVO_4_), emerging as a representative metal oxide photoanode material, has recently attracted intensive interests owing to its suitable band structure and intrinsic electrical properties^11–13^. Nevertheless, BiVO_4_ still suffers from severe bulk charge carrier recombination under solar irradiation conditions due to the low carrier mobility (about 0.044 cm^2^ V^−1^ s^−1^)^12^. The reported photocurrent densities of pure BiVO_4_ are thus much lower than its theoretical value (7.5 mA cm^−2^ under AM 1.5G illumination). Developing strategies to design efficient BiVO_4_ photoelectrodes by addressing its intrinsic drawbacks of poor charge transport has thus been a great challenge.

Various strategies have been developed for improving the charge transport in BiVO_4_ photoanodes such as doping^14,15^, heterostructuring^16–18^, plasmonics^19,20^, and nanostructuring^21^. Among these, metal doping has been used to promote the intrinsic charge transport based on the strengthened lattice distortion. Doping with elements such as W is always accompanied by a sharp decrease of the carrier mobility and carrier lifetime^12^. Mo, however, was reported to be a more efficient electron donor owing to the generated higher density of states^14^ and thus results in much higher electron mobility and photocurrent density than that of W^22^. It can thus be envisaged that a strategy of further improving the intrinsic charge transport on the success of Mo doping represents great advances of addressing the intrinsic charge transport limitations of BiVO_4_.

Forming heterostructures has been found very effective to enhance the charge separation, based on which a remarkable photocurrent density was achieved for a BiVO_4_/WO_3_ photoelectrode by taking the advantages of both photo-active materials^16^. Similarly, a variety of highly conductive materials such as carbon quantum dots^23^, black phosphorus^24^, and graphdiyne^25^, were successfully employed to decorate on the BiVO_4_ surface to enhance the charge transfer. Despite the performance improvements in these rationally designed heterostructures, poor intrinsic carrier transport still hinders further improvement of the BiVO_4_ performance. Inspired by the success of forming heterostructures with highly conductive materials in photoanodes, locating these materials in the bulk instead of the surface of BiVO_4_ photoanode may be a promising strategy for improving its bulk charge transport. However, growing a BiVO_4_ film with these conductive nanoobjects embedded in the matrix seems to be a challenge since BiVO_4_ tends to reject extrinsic materials during crystallization.

In present work, we demonstrate a strategy of embedding laser generated nanocrystals in BiVO_4_ photoanode matrix for enhanced bulk charge transport. Such embedding was found unique owing to the advantage of laser synthesis and processing of colloids (LSPC) for generating well-dispersed nanocrystals in desired solvents. By taking La:BaSnO_3_ (LBSO) that has carrier mobility over 300 cm^2^ V^−1^ S^−1^ (4–5 orders of magnitude higher than that of Mo:BiVO_4_)^26^ as an example, we show in present work that such a strategy could lead to pronounced improvement of photocurrent density up to 5.15 mA cm^−2^ at 1.23 V_RHE_ (from original 4.01 mA cm^−2^). Embedding of other laser generated nanocrystals in the photoanode was found to universally enhance the PEC performance of BiVO_4_, thus providing an effective alternative for addressing the charge transport limitations in metal oxide based photoelectrodes.

## Results

### Laser generated nanocrystals in BiVO_4_ photoanode matrix

**Fig. 1:**
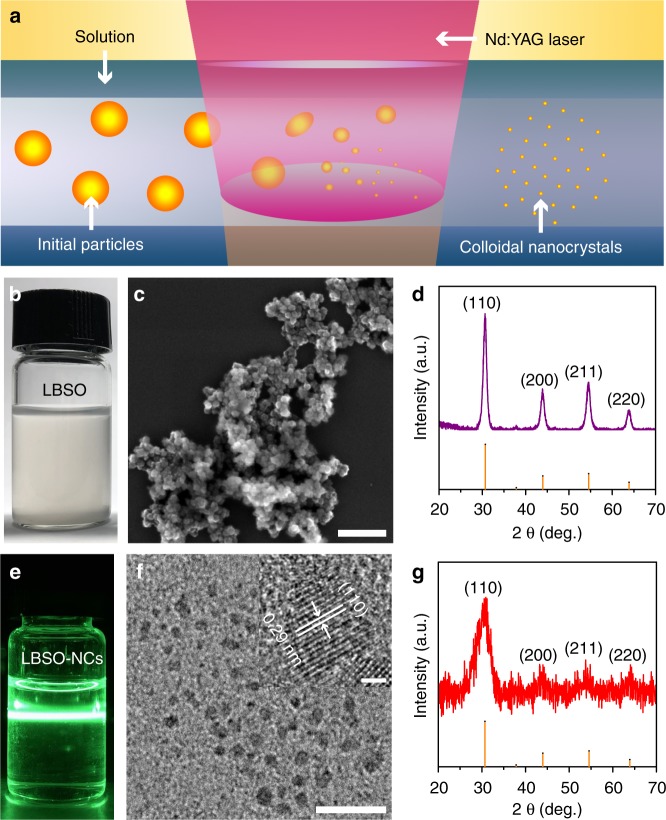
LBSO before and after LSPC. **a** Schematic illustration of LSPC. **b** Optical image of LBSO particles dispersed in the solvent before LSPC. **c** SEM image and **d** XRD pattern of LBSO particles fabricated. **e** Mie-scattering image of laser generated LBSO colloidal nanocrystals (LBSO-NCs). **f** TEM and HRTEM image (insert) of LBSO nanocrystals generated by pulsed laser irradiation. **g** XRD pattern of LBSO nanocrystals after pulsed laser irradiation. Scale bars: (**c**) 250 nm, (**f**) 20 nm and insert of (**f**) 2 nm

Figure 1a schematically illustrates the fabrication of the ligand free LBSO nanocrystals by LSPC^27^, which has so far rarely involved the synthesis of multinary metal oxides^28^. The raw material of LBSO synthesized by a previously reported method^26^ was dispersed by sonication in mixed solvents of water, ethylene glycol, and glacial acetic acid (see Fig. 1b) that were used in the precursor of Mo:BiVO_4_ (MBVO) film preparation. As seen in Fig. 1c and d, the raw LBSO nanoparticles have an average diameter of about 40 nm and cubic crystal phase (PDF card number: 15-0780). The colloidal solution was then illuminated by an unfocused pulsed laser beam with a laser fluence of 382 mJ pulse^−1^ cm^−2^ (third harmonic) for 10 min at room temperature. Subsequently, a transparent colloidal solution that shows Mie-scattering was obtained (Fig. 1e). The TEM image shown in Fig. 1f demonstrates that the size of the laser generated particles decreases from the original 40 nm to less than 10 nm on average. Furthermore, high-resolution TEM (HRTEM) shown in insert of Fig. 1f shows the labeled lattice fringes (0.29 nm) that correspond to the lattice spacing of the perovskite BaSnO_3_ (110) plane. Raman spectroscopy (Supplementary Fig. 1) revealed the same peak positions of the nanocrystals as the raw nanoparticles, indicating a stable crystal structure of LBSO upon pulsed laser irradiation. In addition, the laser generated nanocrystals demonstrated a similar XRD pattern as that of the raw material (Fig. 1d), as shown in Fig. 1g. We can also observe the obvious peak broadening in the XRD pattern of the laser generated nanocrystals, e.g., at around 30.7°, indicating a decrease in crystal size after laser irradiation. Further calculations from the XRD pattern based on the Scherrer equation reveal that the laser generated nanocrystals have an average size of 4.1 nm, which agrees with that shown in the HRTEM observation (Fig. 1f). In comparison to the well-known wet chemical synthesis of inorganic nanocrystals, the technique of LSPC has the advantage of producing nanocrystals without ligand protection that is specifically favorable for optoelectronic applications^29–31^.

**Fig. 2:**
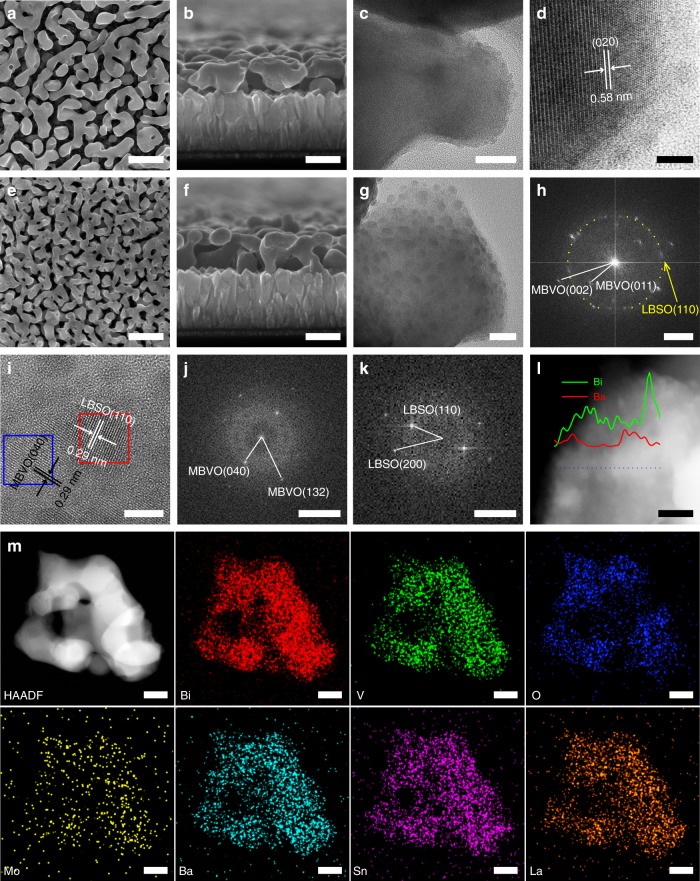
Characterizations of MBVO and LBSO-MBVO films. **a** SEM top-view, **b** SEM cross-section, **c** TEM, and **d** HRTEM image of MBVO films. **e** SEM top-view, **f** SEM cross-section, **g** TEM image, **h** FFT-transformation image and **i** HRTEM image of LBSO-MBVO-2 film. **j** FFT-transformation image of MBVO matrix (area labeled in blue in Figure **i**). **k** FFT-transformation image of LBSO nanocrystal (area labeled in red in Figure **i**). **l** HAADF image and corresponding EDX-line scanning of LBSO-MBVO-2 film. **m** TEM-EDS analysis of LBSO-MBVO-2 film. Scale bars: (**a**, **e**) 500 nm, (**b**, **f**) 250 nm, (**c**, **g**) 20 nm, (**d**, **i**) 5 nm, (**h**) 2 1/nm, (**j**, **k**) 5 1/nm, (**l**) 10 nm and (**m**) 100 nm

We thus tried to introduce the laser generated LBSO nanocrystals with different concentrations (0.137, 0.274, and 0.548 at.%) (Supplementary Fig. 2) in MBVO films, which is denoted as LBSO-MBVO-1, LBSO-MBVO-2, and LBSO-MBVO-3, respectively. The procedures of MBVO photoanode film fabrication and how laser generated LBSO nanocrystals are introduced into the photoanode are depicted in Supplementary Fig. 3. In brief, MBVO films were synthesized according to the reported method^32^, and the LBSO-MBVO films were synthesized by adding the laser generated LBSO nanocrystals in the MBVO precursor while keeping other fabrication conditions unchanged. All films were prepared on fluorine-doped SnO_2_ (FTO) glass substrates followed by an annealing step in air at 500 °C. As shown in Fig. 2a and b, the prepared MBVO films exhibit worm-like nanoporous structure with average diameters below 200 nm. The film thickness is about 250 nm. TEM and TEM-EDS analyses (Fig. 2c and Supplementary Fig. 4) reveal the uniform distribution of the elements Bi, V, O, and Mo throughout the MBVO film. The HRTEM image shown in Fig. 2d reveals a d-spacing of 0.58 nm, corresponding to the monoclinic BiVO_4_ (020) plane.

X-ray diffraction (XRD, Supplementary Fig. 5a) shows that the crystal structure of the MBVO is not significantly affected by the incorporation of LBSO nanocrystals, and no obvious diffraction peaks deviation was found indicating the negligible influence on the crystal structure of MBVO films. The UV-vis spectra shown in Supplementary Fig. 5b indicates little differences between MBVO film and LBSO-MBVO films, except the slight increase at wavelength shorter than 450 nm, probably due to the introduced LBSO. Morphologically, the nanoporous structure of the LBSO-MBVO film becomes much denser and smaller upon increasing the LBSO concentration. Too much LBSO incorporation would result in the formation of an inhomogeneous film (Supplementary Fig. 6). Figure 2e shows a typical SEM image of the LBSO-MBVO film and illustrates the obvious decrease in size of the worm-like structures. The film thickness (Fig. 2f) still remains the same (about 250 nm), which is reasonable in view of the unchanged precursor concentrations. The TEM image shown in Fig. 2g reveals that the LBSO-MBVO film is decorated with nanocrystals with the size of about 5–10 nm, representing a clear difference with the unmodified MBVO film (Fig. 2c). Further characterization reveals that the nanocrystals distributed in the MBVO lattice background demonstrated obviously different contrast/lattice-fringes from the matrix (see Supplementary Fig. 7). As shown in the corresponding FFT-transformation of the area shown in Fig. 2g (Fig. 2h), the MBVO matrix can be determined by labeling the (011) and (002) plane and their angle of 23.3° (see calculation in ‘Methods'' section). The distributed LBSO can be determined by the labeled diffraction ring that is composed of many prolonged diffraction dots, which is corresponding to the crystal plane of (110) of LBSO with the inter-planar distances of 0.29 nm. The polycrystalline feature from the diffraction ring is also in agreement with their random distribution in the matrix (see Supplementary Fig. 7). Figure 2i shows the typical crystal lattice of the MBVO matrix and the distributed LBSO nanocrystal. The corresponding FFT-transformations were further carried out in the labeled areas shown in Fig. 2i to distinguish the crystal lattice between the matrix and the embedded nanocrystal. The result of the labeled blue area and red area are shown in Fig. 2j and k, respectively, which determines the MBVO lattice matrix by labeling the (040) and (132) plane and their angle of 59.7°, as well as the lattice of the embedded LBSO by labeling the (110) and (200) plane with the angle of 45°. In addition, we have also observed in the edge area of the MBVO matrix the signal rising of the elemental Ba and Sn in the nanocrystal areas along the EDX-line scanning (Fig. 2l and Supplementary Fig. 8), which further implies the embedding of LBSO nanocrystals in MBVO matrix. TEM-EDS mapping (Fig. 2m) was used to analyze the overall distribution of LBSO in the MBVO matrix, which confirms that the LBSO nanocrystals are homogeneously embedded throughout the MBVO matrix. This is different from the typical core-shell distribution that is often observed for surface decorated nanostructures^33–35^.

As far as we know, there have been rare reports on such well-embedded nanocrystals in BiVO_4_ matrix. The uniquely well-dispersed LBSO nanocrystals in the precursor solvent presumably serves as the nucleus for BiVO_4_ growth, which may explain the intimate embedding of the LBSO nanocrystals in the MBVO matrix. It should be noted that generating well-dispersed nanocrystals in complicated solvents (Fig. 1f and Supplementary Fig. 9) would be a challenge for conventional wet chemical synthesis (CWCS), owing to the limitation of CWCS for producing ligand-free nanocrystals in desired solvent, as well as the lack of strategies for producing multinary metal oxide based nanocrystals. To further demonstrate the unique advantage of LSPC for embedding the nanocrystals in photoanode, we tried to sonicate an equivalent amount of raw LBSO particles in the precursor solvents (the procedure is described in Supplementary Fig. 10). It turns out that few nanocrystals were found in the MBVO matrix, and inhomogeneous films with many large LBSO agglomerates on the surface were obtained. This always resulted in poorer PEC performance compared to the unmodified MBVO films (Supplementary Fig. 11). The relatively large average size (around 40 nm) and easy aggregation of LBSO raw particles in the mixed solvents (shown in Fig. 1c), even after sonication treatment, rendered them easily spun away during the film preparation with a high spinning speed (3000 rpm). We have also prepared LBSO particles with larger sizes (hundreds of nanometers, Supplementary Fig. 12a) and tried to embed them in the MBVO matrix. However, due to the high spinning speed (3000 rpm) used for the MBVO film preparation (such speed would spin away the large particles from the film), very few LBSO particles were found in the films (Supplementary Fig. 12c and d), which resulted always in unimproved PEC performance (Supplementary Fig. 12b).

### PEC water splitting performance of LBSO-MBVO films

**Fig. 3:**
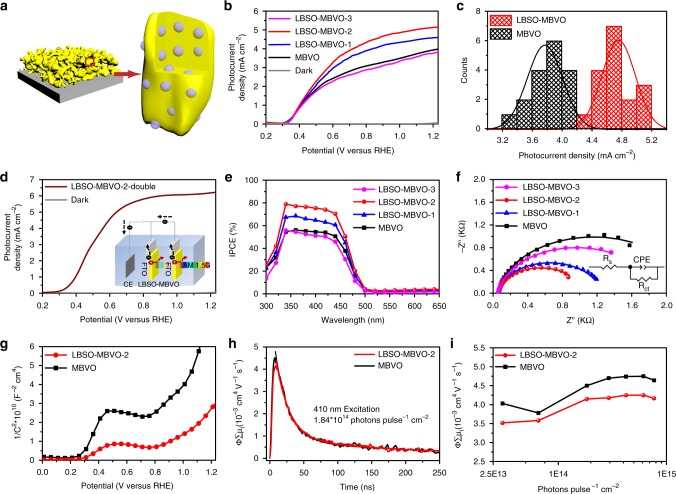
Performance of MBVO and LBSO-MBVO films. **a** Schematic representation of pulsed laser generated nanocrystals embedded MBVO films. **b** J–V curves of different LBSO-MBVO photoanodes under AM 1.5G irradiation. **c** Photocurrent density distribution of MBVO and LBSO-MBVO films at 1.23 V_RHE_. **d** J–V curves of the LBSO-MBVO-2 dual photoanodes under AM 1.5G irradiation. Insert: schematic of LBSO-MBVO-2 photoanodes with dual configuration. **e** IPCE curves at 1.23 V_RHE_ and **f** EIS curves of different LBSO-MBVO photoanodes. **g** MS curves of MBVO and LBSO-MBVO-2 films. **h** Time-resolved microwave conductance signals recorded for MBVO and LBSO-MBVO-2 films using a 410 nm laser pulse with a photon flux of 1.84 × 10^14^ photons pulse^−1^ cm^−2^. **i** Maximum TRMC signals as a function of incident photons per laser pulse for MBVO and LBSO-MBVO-2 films

To evaluate how the embedding of LBSO nanocrystals (Fig. 3a) impacts the PEC water splitting performance of MBVO films, the PEC measurements were conducted using cyclic voltammetry (CV) in a three-electrode cell with a scan rate of 10 mV s^−1^ under AM 1.5G illumination (100 mW cm^−2^). A 0.1 M Na_2_SO_3_ aqueous solution was used as the electrolyte, buffered with a 1.0 M potassium phosphate buffer (pH = 7). Figure 3b shows the photocurrent density–potential (J–V) curves of MBVO and different LBSO-MBVO champion films. The MBVO photoanode exhibits a top photocurrent density of 4.01 mA cm^−2^ at 1.23 V_RHE_ under front illumination. After embedding of LBSO nanocrystals into the matrix, the photocurrent density reaches a top value of 5.15 mA cm^−2^ at 1.23 V_RHE_ for the LBSO-MBVO-2 sample. However, further increasing the concentration of LBSO colloidal solution results in a decrease of the photocurrent density, which is due to the poor homogeneity of the films (Supplementary Fig. 6d). To take an overview of the effect of LBSO nanocrystals on MBVO PEC performance, the histograms of more than 30 MBVO (deviation of 6.1%, Supplementary Fig. 13) and LBSO-MBVO films (deviation of 4.6%, Supplementary Fig. 13) are shown in Fig. 3c. It can be seen that LBSO nanocrystals embedded MBVO films could reach an average photocurrent density of 4.76 mA cm^−2^, while that of unmodified MBVO films was only 3.77 mA cm^−2^ at 1.23 V_RHE_. Based on LBSO-MBVO-2 film, we further assembled dual photoanodes. Its champion photocurrent density could reach 6.22 mA cm^−2^ at 1.23 V_RHE_ (Fig. 3d), which is among the top in the performance records of BiVO_4_ based photoanodes (Supplementary Table 1). The photocurrent densities were also measured in aqueous solution without the Na_2_SO_3_ hole scavenger (Supplementary Fig. 14). It can be seen that the control film can reach 2.07 mA cm^−2^ at 1.23 V_RHE_, while it increases to 2.38 and 3.23 mA cm^−2^ for the LBSO-MBVO-1 and LBSO-MBVO-2 films respectively. When further increasing the concentration of the LBSO colloidal solution, the photocurrent intensity of the resulted film (LBSO-MBVO-3) dropped to 1.87 mA cm^−2^, which is lower than that of the control film. It should be mentioned that the tendency of the photocurrent intensities evolution shown in Supplementary Fig. 14 is similar with those measured in the electrolyte with Na_2_SO_3_. Incident photon-to-current conversion efficiency (IPCE) measurements were performed at 1.23 V_RHE_. The photocurrent response spans a wavelength region from 300 nm to 500 nm, and the champion efficiency of LBSO-MBVO-2 film could reach up to 80%. In contrast, the IPCE of the MBVO film is lower than 55% through the entire responsive region (Fig. 3e).

The bulk charge separation efficiencies can be further calculated by the photocurrent density and absorbance, and η_bulk_ of LBSO-MBVO-2 films reach up to 87% while that of MBVO film is only about 65% (Supplementary Fig. 15), indicating the facilitation of the charge separation by embedding LBSO nanocrystals. Electrochemical impedance spectroscopy (EIS) and Mott-Schottky (MS) measurements were used to further investigate electronic properties in different films. As EIS results shown in Fig. 3f, by using the equivalent circuit model, we can easily obtain the R_s_ and R_ct_ value, which represents the series resistance and charge transfer resistance, respectively^36,37^. MBVO and LBSO-MBVO films exhibit similar R_s_ values, while a much smaller R_ct_ of LBSO-MBVO-2 film is obtained (979.5 Ω) compared to MBVO films (2199 Ω) (Supplementary Table 2), indicating the enhanced charge transfer by introducing of LBSO nanocrystals. The most likely explanation is that the embedded LBSO nanocrystals is probably a better OER catalyst than the BVO itself. Mott-Schottky (MS) curves (Fig. 3g) reveal that introducing the LBSO nanocrystals does not change the n-type feature and flat band potential of MBVO films, but resulted in much shallower slopes, indicating higher carrier densities. The values of carrier densities are based on the calculation according to Equation 8 and listed in Supplementary Table 4, which also clearly shows multiple increase of carrier densities. It should be noted that increasing the concentration of LBSO colloidal solutions results in an increase of the carrier density (Supplementary Fig. 16 and Supplementary Table 4), while the photocurrent density after incorporation of nanocrystals does not always increase. This is probably due to the formation of an inhomogeneous film when adding too much LBSO nanocrystals (Supplementary Fig. 6).

To further investigate the bulk charge transport property, time-resolved microwave conductivity (TRMC) of MBVO and LBSO-MBVO-2 films were measured, and the results are shown in Fig. 3h and i. The mobility of the sample can be determined from the peak amplitude of the microwave transient signal, and the diffusion length can be calculated based on the lifetime obtained from an exponential fit of the transient. As shown in Supplementary Table 3, the MBVO and LBSO-MBVO-2 films showed modest TRMC mobility with only slight variations, which is out of our expectation that the embedding of LBSO that has carrier mobility over 300 cm^2^ V^−1^ S^−1^ (4–5 orders of magnitude higher than that of Mo:BiVO_4_) is more likely capable of increasing the carriers mobility of the MBVO-LBSO film. This suggests that the photogenerated carriers in MBVO are not injected into the LBSO nanocrystals. When plotting the TRMC mobility as a function of light intensity, the values increase with increasing photon flux for samples MBVO and LBSO-MBVO-2. This indicates a large density of traps (“trap filling effect”), which are similar with the previous observations^12^. The large density of traps indicated by the mobility increase upon increasing photon flux could be due to the Mo doping, and also could be due to the embedding of LBSO nanocrystals into the matrix, which generates much LBSO-BVO interfaces that would trap the carriers. It should be mentioned that a decreased mobility upon doping or sample modification does not necessarily imply a reduced photocurrent^12^, but probably hints towards a charge transport model through polaron hoping characterized by a fast trapping, i.e., “loss” in mobility, and enhanced formation of polarons^38^. However, if and how the embedding of LBSO in the MBVO matrix influence the formation of polarons is still not yet clear and will be investigated in our future work. As TRMC results confirm the little variations of carrier mobility and lifetime upon LBSO embedding, the embedding of LBSO would lead to enhanced electronic conductivity owing to the increased carrier densities reflected by the above MS data (Fig. 3g), which is similar with the cases of W doping in BiVO_4_ film^8,12,39^.

## Discussion

**Fig. 4:**
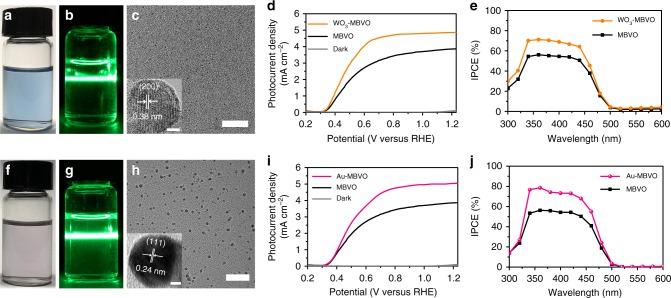
Characterizations of WO_3_ and Au nanocrystals and performance of WO_3_-MBVO and Au-MBVO films. **a** Optical image of laser generated WO_3_ colloidal nanocrystals with the mixed solvents. **b** Mie-scattering images of laser generated WO_3_ colloidal nanocrystals. **c** TEM and HRTEM images of laser generated WO_3_ nanocrystals. **d** J–V and **e** IPCE curves of MBVO and WO_3_-MBVO photoanodes. **f** Optical image of laser generated Au colloidal nanocrystals with the mixed solvents. **g** Mie-scattering images of laser generated Au colloidal nanocrystals. **h** TEM and HRTEM images of laser generated Au nanocrystals. **i** J–V and **j** IPCE curves of MBVO and Au-MBVO photoanodes. Scale bars: (**c**, **h**) 50 nm and insert of (**c**, **h**) 2 nm

We further found that embedding nanocrystals in MBVO matrix is a general strategy for boosting its photoelectrochemical performance. We similarly prepared sub-10-nm WO_3_ and Au nanocrystals^40^ in the precursor solvents through the LSPC (Fig. 4a–c and f–h), and then introduced these ligand-free nanocrystals into the MBVO photoanode. The embedding structure was verified by the TEM-EDS characterizations (Supplementary Figs. 17 and 18). Analysis of XRD and UV-Vis of WO_3_-MBVO film results indicates almost no change in films structure upon the nanocrystals embedding (Supplementary Fig. 19). Analysis of XRD results of Au-MBVO film indicates almost no change in films structure upon embedding of the nanocrystals, while UV-Vis results show an obvious absorption increase at a wavelength of about 480 nm (Supplementary Fig. 20), which is probably due to the plasmonic effect of the Au nanocrystals. The photoelectrochemical measurements revealed that embedding WO_3_ and Au nanocrystals in MBVO matrix could lead to the enhancement of photocurrent density up to 4.86 and 5.05 mA cm^−2^ at 1.23 V_RHE_, respectively (Fig. 4d and i). The WO_3_-MBVO and Au-MBVO films also demonstrate obvious enhancement in IPCE spectra in the wavelength range from 300 nm to 500 nm (Fig. 4e and j). Similarly, the EIS, MS, and TRMC analysis revealed that embedding of WO_3_ and Au nanocrystals resulted in the enhancement of the charge transfer, increase of the charge carrier densities, and little variations on carrier mobility and lifetime, which are all in agreement with the case of embedding LBSO nanocrystals into the MBVO matrix (Supplementary Figs. 21 and 22, Supplementary Tables 5–10). These results thus provide us plenty of room for modulating BiVO_4_ performance by embedding nanocrystals with tailored physical/chemical properties.

In summary, we have demonstrated an efficient and general strategy for boosting BiVO_4_ based photoanode performance by embedding laser generated nanocrystals in its matrix. Such embedding was found unique owing to the advantages of LSPC for producing ligand free nanocrystals in any desired solvents. TRMC results confirm the little variations of carrier mobility and lifetime upon LBSO embedding. The embedding of LBSO led to enhanced electronic conductivity owing to the increased carrier density. The charge transfer was also favorably affected by the embedding of LBSO nanocrystals. Further exploring of introducing other laser generated nanocrystals in the BiVO_4_ matrix indicates such embedding strategy is general to address the slow bulk charge transport that bothers most metal oxides. The champion photocurrent density of 6.22 mA cm^−2^ at 1.23 V_RHE_ for the LBSO-MBVO photoanodes with a dual configuration as well as the universal feature of boosting the PEC water splitting performance via embedding other ligand free nanocrystals suggest that our study will provide an effective alternative to boost the PEC performance of metal oxide based photoelectrodes.

## Methods

### Materials

Fuorine-doped tin oxide (FTO) coated glass substrates (~30 Ω) were purchased from Pilkington. Bismuth nitrate pentahydrate (Bi(NO_3_)_3_·5H_2_O, >98%), molybdenum diacetylacetonate dioxide (MoO_2_(acac)_2_), tri-block copolymer Pluronic® F-108, ethylene glycol (EG), tungsten(VI) oxide nanopowders (WO_3_, ~100 nm), and Lanthanum(III) nitrate hydrate (La(NO_3_)_3_, 99.9%) were obtained from Sigma-Aldrich and used as received. Vanadium acetylacetone oxygen (VO(acac)_2_, >99%) and glacial acetic acid (>99.7%) were purchased from Acros Organics and Fisher Scientific, respectively. Potassium phosphate monobasic (KH_2_PO_4_, >98%), sodium sulfite (Na_2_SO_3_, >97%), barium dichloride (BaCl_2_, >99.5%), tin(II) chloride (SnCl_2_, >98%), hydrogen peroxide aqueous solution (H_2_O_2_), ammonium hydroxide aqueous solution (NH_4_OH), and sodium hydroxide (NaOH) were purchased from Sinopharm Chemical Reagent Co., Ltd. Au plate was obtained from Zhongnuo Advanced Material (Beijing) Co., Ltd. China. Deionized water was used with a resistivity of 18.25 MΩ·cm.

### Preparation of LBSO particles

The fabrication of La:BaSnO_3_ particles was followed and improved by a solution process developed by Shin^26^. In brief, La:BaSnO_3_ particles were fabricated by reacting of BaCl_2_, SnCl_2_ and La(NO_3_)_3_ in an aqueous solution of H_2_O_2_ and NH_4_OH at 50 °C, and followed by vacuum filtration and sintering at 500 °C for 1 h.

### Laser generated nanocrystals synthesis

A Nd:YAG laser (pulse width 10 ns, repetition rate 10 Hz, beam size 10 mm) was used as the laser source for pulsed laser irradiation. To fabricate La:BaSnO_3_ (LBSO) nanocrystals, 1, 2, and 4 mg of as-received LBSO particles were firstly well dispersed in 4 ml solution (ethylene glycol: glacial acetic acid: deionized water = 3:4:1) by ultrasonic vibration. The mixtures were then irradiated by an unfocused laser beam with a laser fluence of 382 mJ pulse^−1^ cm^−2^ (third harmonic) for 10 min at room temperature. Similarly, 3 mg of commercial WO_3_ particles were dispersed in 4 ml solution mentioned above and irradiated by the unfocused laser beam with a laser fluence of 446 mJ pulse^−1^ cm^−2^ (third harmonic) for 5 min. For Au nanoparticles, the Au plate was first put in the solution and irradiated by 1064 nm with a laser fluence of 1.08 J pulse^−1^ cm^−2^ for 1 min. After that, Au plate was taken out of the solution remaining the solution being irradiated by 1064 nm with a laser fluence of 955 mJ pulse^−1^ cm^−2^ for another 10 min.

### Preparation of MBVO photoanodes

Mo:BiVO_4_ photoanodes were fabricated by the metal organic decomposition (MOD) process and spin-coating deposition via a modified method reported by Nair^32^. Typically, 0.15 M Bi(NO_3_)_3_·5H_2_O as Bi precursor was dissolved in the solvent containing 1.5 ml ethylene glycol, 2 ml glacial acetic acid and 0.5 ml deionized water, stirred for 20 min at room temperature. Then 0.3 M VO(acac)_2_ as V precursor was added subsequently to the above solution, stirred for another 1 h. The incorporation of 2% Mo into the solution was realized by dissolving 50 mM MoO_2_(acac)_2_ into ethylene glycol and taking 240 μL out to add to the solution. The 0.35 g tri-block copolymer F-108 as structural agent was then added in and stirred for 2 h to make a porous structure. Finally, a uniform viscous ink for spin coating was obtained. Mo:BiVO_4_ photoanodes were deposited on cleaned FTO coated glass substrates at 3000 rpm for 50 s followed by baking at 250 °C for 5 min and annealing in a box furnace at 500 °C for 1 hour with a heating rate of 3 °C/min. To obtain a desired film with proper thickness, the spin-coating deposition procedure was repeated thrice. After sintering, 1 M NaOH was used to remove the excess V_2_O_5_.

### Embedding laser generated nanocrystals in MBVO photoanodes

The embedding procedure of laser generated nanocrystals in Mo:BiVO_4_ photoanodes is similar with that of Mo:BiVO_4_ photoanode except the preparation of the precursors. For the case of LBSO-MBVO, 0.15 M Bi(NO_3_)_3_·5H_2_O was firstly dissolved in the solution containing 1.125 ml ethylene glycol, 1.5 ml glacial acetic acid and 0.375 ml deionized water, and then 1 ml of laser-treatment LBSO colloidal solution was added. Then 0.3 M VO(acac)_2_ as V precursor was added subsequently to the above solution, stirred for another 1 hour. The incorporation of 2% Mo into the solution was realized by dissolving 50 mM MoO_2_(acac)_2_ into ethylene glycol and taking 240 μL out to add to the solution. The 0.35 g tri-block copolymer F-108 as structural agent was then added in and stirred for 2 h. 0.137% LBSO-Mo:BiVO_4_ (LBSO-MBVO-1), 0.274% LBSO-Mo:BiVO_4_ (LBSO-MBVO-2) and 0.548% LBSO-Mo:BiVO_4_ (LBSO-MBVO-3) (at.%) were obtained by choosing different laser treated LBSO colloidal solution concentrations. In the similar way, WO_3_-Mo:BiVO_4_ (WO_3_-MBVO) and Au-Mo:BiVO_4_ (Au-MBVO) were fabricated.

### Characterization

The crystal structures of films were characterized using X-ray diffraction spectra (XRD, X’pert PRO, Panalytical) with Cu Kα (λ = 0.15406 nm) radiation at room temperature. Grazing incidence XRD measurements with incidence angle of 1 degree was used. The film morphologies were identified using a field emission scanning electron microscope (FESEM). High-resolution transmission electron microscopy (HRTEM) was performed in combination with EDS using a FEI Tecnai F30 microscope equipped with a field emission gun (FEG) operated at 300 kV, a high angle annular dark field (HAADF) STEM detector and an Oxford Instruments EDS detector. The absorbance was measured using the ultraviolet-visible (UV-vis) spectrophotometer (Perkin-Elmer Lambda 35 UV-vis-NIR). The Raman spectra were obtained using a Renishaw in Via Raman microscope with a 532 nm laser.

### Photoelectrochemical measurements

The photoelectrochemical measurements were all carried out using an electrochemical workstation (CHI660E) and a three-electrode cell with a flat quartz window to facilitate the light to pass through at room temperature. The photoelectrodes mentioned in this work were used as working electrodes while Ag/AgCl electrode and Pt electrode were used as reference and counter electrodes, respectively. The light source was a Xe 500 W lamp (CEL-S500, CEAULIGHT) with an AM 1.5G filter (100 mW cm^−2^). Typically, the exposed area of the photoelectrode was 0.26 cm^2^. 1 M potassium phosphate buffer with and without 0.1 M Na_2_SO_3_ aqueous solution (pH = 7) were used as the electrolyte. J–V curves were recorded in the anodic direction with the potential range from −0.6 to 0.8 V vs. Ag/AgCl and the scan rate for the measurements was 10 mV s^−1^. The incident photon to current efficiency (IPCE) measurements were performed using the same three-electrode cell described above. The simulated illumination source was consisted of a full solar simulator (CEL-S500, CEAULIGHT) and a motorized monochromator (CEL-IS302, CEAULIGHT). An applied potential of 1.23 V_RHE_ was supplied by a CHI760E electrochemical workstation and the power density at every specific wavelength was measured by a CEAULIGHT CEL-NP2000-2 power meter. Electrochemical impedance spectroscopy (EIS) spectra were collected under illumination of AM 1.5G simulated solar light (100 mW cm^−2^) at 1.23 V_RHE_ in 1.0 M potassium phosphate buffer (frequency range: 0.1 Hz–100 kHz). Mott-Schottky (MS) spectra were obtained in the voltage window of 0~1.23 V_RHE_ in 1.0 M potassium phosphate buffer (increment: 5 mV, frequency: 1 kHz).

### Time-resolved microwave conductivity (TRMC) measurements

TRMC measurements were carried out by placing the sample in a microwave cavity cell based on the setup described elsewhere^41,42^. The signal of change in the microwave power (ΔP/P) was reflected by the cavity upon the sample excitation by a Q-switched Nd:YAG laser at the wavelength of 410 nm and is correlated to the photoinduced change in the conductance of the sample (ΔG), which can be denoted by:

1 $${\mathrm{\Delta P}}/{\mathrm{P}}({\mathrm{t}}) = - {\mathrm{K\Delta G}}({\mathrm{t}})$$

where K is the sensitivity factor derived from the resonance characteristics of the cavity and the dielectric properties of the medium (ε_r_ of BiVO_4_ is taken as 68 F m^−1^)^37^. The photoinduced change in the conductance of the sample further correlates to the product of the charge carrier generation yield (ϕ) and the sum of electron and hole mobilities (Σμ), which can be obtained by

2 $${\mathrm{\phi}} {\mathrm{\Sigma \mu }} = {\mathrm{\Delta G}}/\left( {{\mathrm{I}}_0{\mathrm{\beta eF}}_{\mathrm{A}}} \right)$$

where I_0_ is the incident intensity per pulse, e is the elementary charge, β is the ratio between the inner broad and narrow dimensions of the waveguide, and F_A_ is the fraction of incident photons absorbed within the sample. The laser pulse intensities were adjusted by the use of calibrated filters and varied from 10^13^ to 10^15^ photons cm^−2^ pulse^−1^.

### Calculations

The recorded potentials versus Ag/AgCl (E_Ag/AgCl_) were converted against reversible hydrogen electrode (RHE) using the Nernst equation^43^:

3 $${\mathrm{E}}_{{\mathrm{RHE}}} = {\mathrm{E}}_{{\mathrm{Ag}}/{\mathrm{AgCl}}} + 0.0591\,{\mathrm{V}} \times {\mathrm{pH}} + {\mathrm{E}}_{{\mathrm{Ag}}/{\mathrm{AgCl}}}^{\mathrm{\theta }}$$

where E_Ag/AgCl_ represents the potential vs. Ag/AgCl, the value of ${\mathrm{E}}_{{\mathrm{Ag}}/{\mathrm{AgCl}}}^{\mathrm{\theta }}$ is 0.197 V_NHE_ at room temperature and E_RHE_ represents the potential vs. RHE.

Incident-photon-to-current conversion efficiency (IPCE) values can be expressed as the following equation^44^:

4 $${\mathrm{IPCE(\% )}} = \left( {{\mathrm{J}} \,\times 1240} \right)/\left( {{\mathrm{\lambda }} \times {\mathrm{P}}_{{\mathrm{light}}}} \right) \times 100\%$$

where J represents the photocurrent density (mA cm^−2^), λ and P_light_ are the incident light wavelength (nm) and the corresponding power density (mW cm^−2^), respectively.

Light harvesting efficiency (LHE) can be expressed as equation as follows^43^:

5 $${\mathrm{LHE}} = 1 - 10^{ - {\mathrm{A}}({\mathrm{\lambda }})}$$

where A(λ) represents absorbance, λ is wavelength. The maximum photocurrent density (J_max_) can be calculated as follows: For the first step, converting the solar spectral irradiance at AM 1.5G (NREL, radiation energy (W m^−2^ nm^−1^) vs. wavelength (nm)) to the solar energy spectrum in terms of number of photons (s^−1^ m^−2^ nm^−1^) vs. wavelength (nm) is necessary. Then, J_max_ is calculated and converted from the number of photons above the photo-active range of the BiVO_4_. J_abs_ (assuming 100% APCE) can be expressed using the following equation^43^:

6 $${\mathrm{J}}_{{\mathrm{abs}}} = {\mathrm{J}}_{{\mathrm{max}}} \times {\mathrm{LHE}}$$

η_bulk_ can be determined by equation as follows:

7 $${\mathrm{\eta }}_{{\mathrm{bulk}}} = {\mathrm{J}}\left( {{\mathrm{Na}}_2{\mathrm{SO}}_3} \right)/{\mathrm{J}}_{{\mathrm{abs}}}$$

where J (Na_2_SO_3_) is the photocurrent density tested in 1 M potassium phosphate buffer with 0.1 M Na_2_SO_3_ (pH = 7).

The donor concentration (N_d_) is calculated according to the MS curves by the following equation^37^:

8 $${\mathrm{N}}_{\mathrm{d}} = (2/{\mathrm{e\varepsilon }}_0{\mathrm{\varepsilon }}) \, \times \left( {{\mathrm{d}}\left( {1/{\mathrm{C}}^2} \right)/{\mathrm{dV}}_{\mathrm{s}}} \right)^{ - 1}$$

where e (electronic charge) is 1.602 × 10^−19^ C, ε_0_ (vacuum permittivity) is 8.854 × 10^−12^ F m^−1^, and ε (relative permittivity) is 68 F m^−1^ for BiVO_4_^37^. C (the space charge capacitance, F cm^−2^), V_s_ (the applied potential, V) and d (1/C^2^)/dV_s_ are obtained from MS curves.

Crystallite size analysis of XRD patterns used the Scherrer equation:

9 $${\mathrm{D}} = {\mathrm{K\lambda }}/\left( {{\mathrm{\beta cos}}\,{\mathrm{\theta }}} \right)$$

where D is crystallite size, K is the Scherrer constant (0.89, assuming spherical particles), λ is the X-ray wavelength (0.15406 nm), θ is the scattering half-angle (15.344°) and β is the FWHM of the peak in the sample pattern (2.0°).

Calculation of the included angle of BiVO_4_ crystal plane (monoclinic):

10 $${\mathrm{cos\theta }} = {\mathrm{d}}_1{\mathrm{d}}_2/{\mathrm{sin}}^2{\mathrm{\beta }}\left[ {{\mathrm{h}}_1{\mathrm{h}}_2/a^{2 + }{\mathrm{k}}_1{\mathrm{k}}_2{\mathrm{sin}}^2{\mathrm{\beta }}/{\mathrm{b}}^2 + {\mathrm{l}}_1{\mathrm{l}}_2/{\mathrm{c}}^2-\left( {{\mathrm{l}}_1{\mathrm{h}}_2 + {\mathrm{l}}_2{\mathrm{h}}_1} \right){\mathrm{cos\beta }}/{\mathrm{ac}}} \right]$$

where θ is the included angle; a, b, c and β are crystal lattice parameters; h_1_, k_1_, l_1_, h_2_, k_2_ and l_2_ are specific crystal indices.

Calculation of the included angle of BaSnO_3_ crystal plane (cubic):

11 $${\mathrm{cos\theta }} = \left( {{\mathrm{h}}_1{\mathrm{h}}_2 + {\mathrm{k}}_1{\mathrm{k}}_2 + {\mathrm{l}}_1{\mathrm{l}}_2} \right)/\sqrt {\left( {{\mathrm{h}}_1^2 + {\mathrm{k}}_1^2 + {\mathrm{l}}_1^2} \right)\left( {{\mathrm{h}}_2^2 + {\mathrm{k}}_2^2 + {\mathrm{l}}_2^2} \right)}$$

where θ is the included angle; a, b, c, and β are crystal lattice parameters; h_1_, k_1_, l_1_, h_2_, k_2_ and l_2_ are specific crystal indices.

## Supplementary information

Supplementary Information

## Data Availability

The authors declare that the data supporting the findings of this study are available within the article and its Supplementary Information files.
