# Supplementary material for: Embedding laser generated nanocrystals in BiVO4 photoanode for efficient photoelectrochemical water splitting
Source: Nat Commun. 2019 Jun 13;10:2609. doi: 10.1038/s41467-019-10543-z (PMC6565742; doi:10.1038/s41467-019-10543-z)
Supplement: Supplementary file 1 — Supplementary Information [file 41467_2019_10543_MOESM1_ESM.pdf]

Supplementary Information

Embedding Laser Generated Nanocrystals in $BiVO_4$ Photoanode for Efficient Photoelectrochemical Water Splitting

Jian et al.

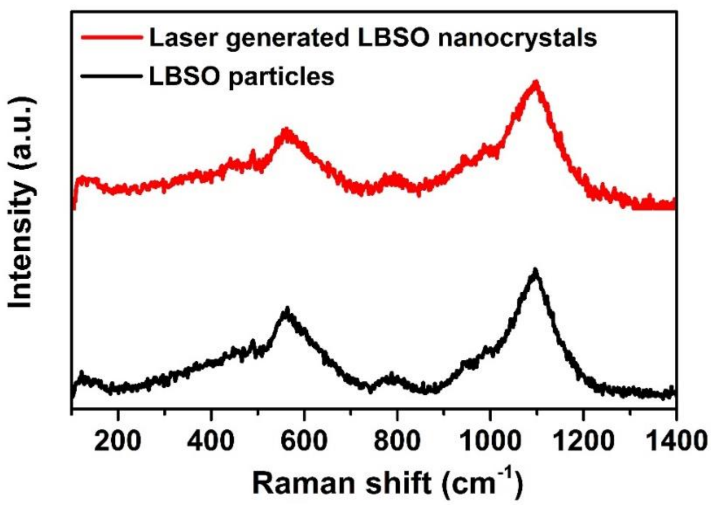

Supplementary Figure 1. Raman spectra of raw LBSO particles and laser generated LBSO nanocrystals.

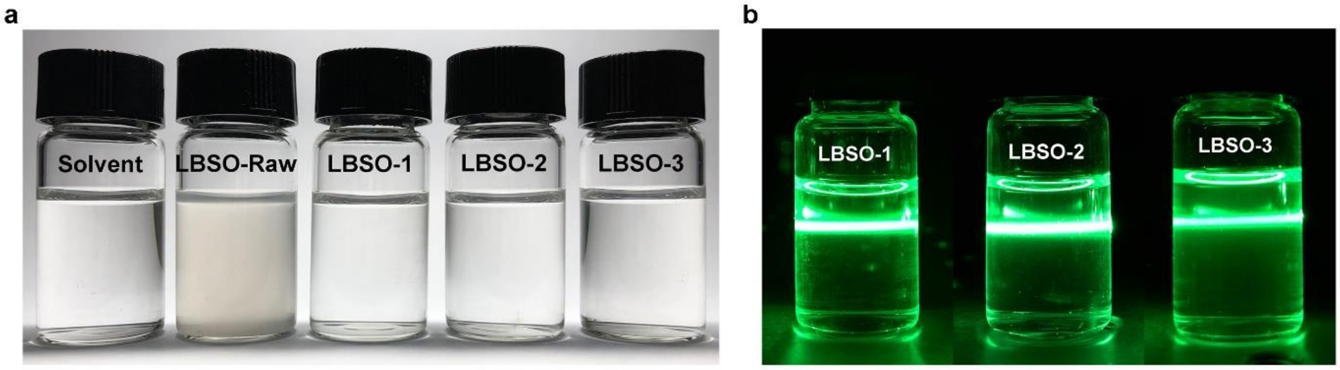

Supplementary Figure 2. Photographs of LBSO in the mixed solvent before and after LSPC. (**a**) Optical images of the mixed solvent (water, ethylene glycol, and glacial acetic acid), LBSO particles simply dispersed in the mixed solvent by sonication, and different laser generated LBSO colloidal nanocrystals. (**b**) Mie-scattering images of different laser generated LBSO colloidal nanocrystals.

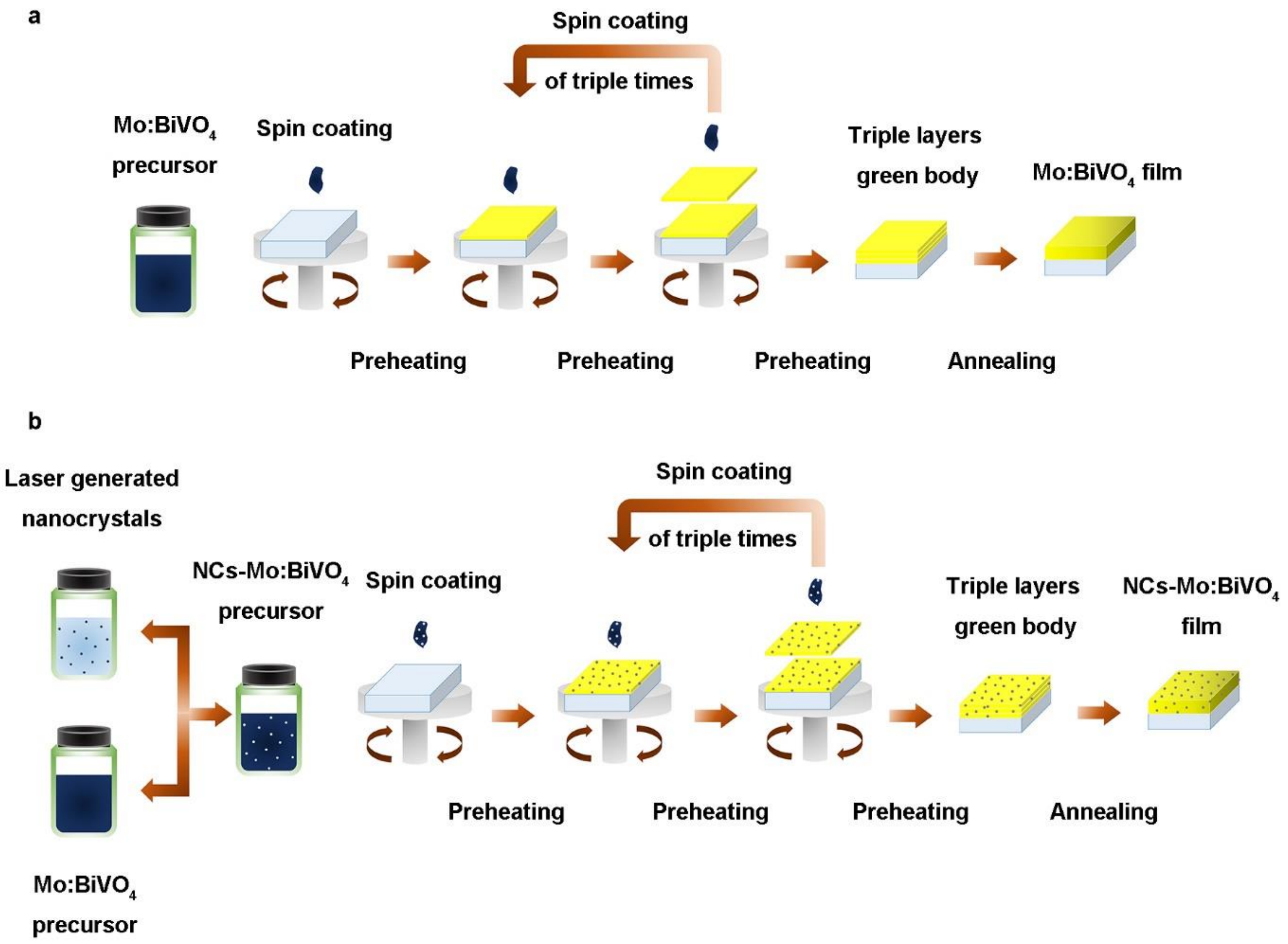

Supplementary Figure 3. Schematic illustration of the fabrication procedures. (**a**) Mo:$BiVO_4$ photoanodes and (**b**) NCs-Mo:$BiVO_4$ photoanodes.

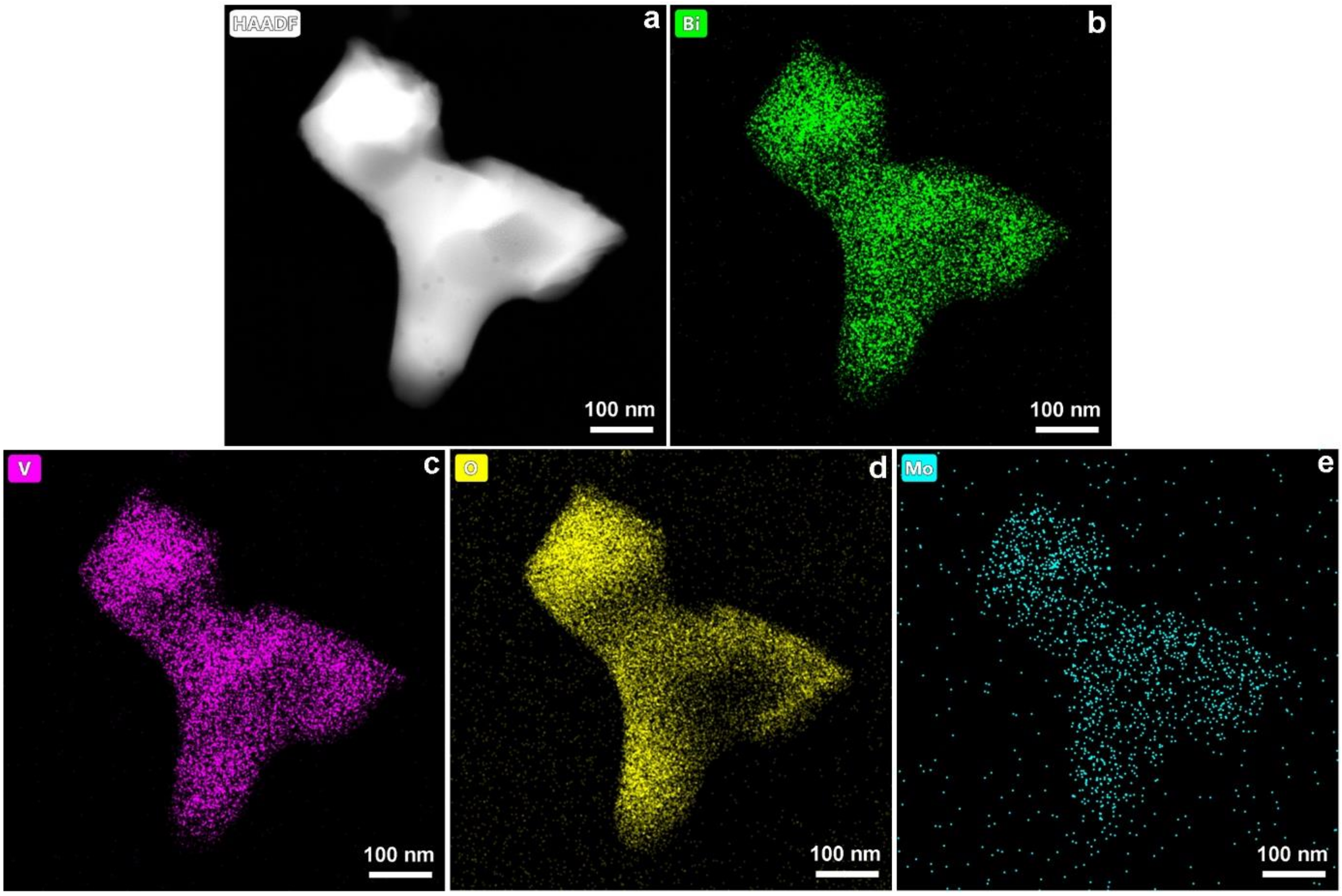

Supplementary Figure 4. TEM-EDS analysis of MBVO film. (**a**) HAADF image, (**b**) Bi, (**c**) V, (**d**) O and (**e**) Mo mapping.

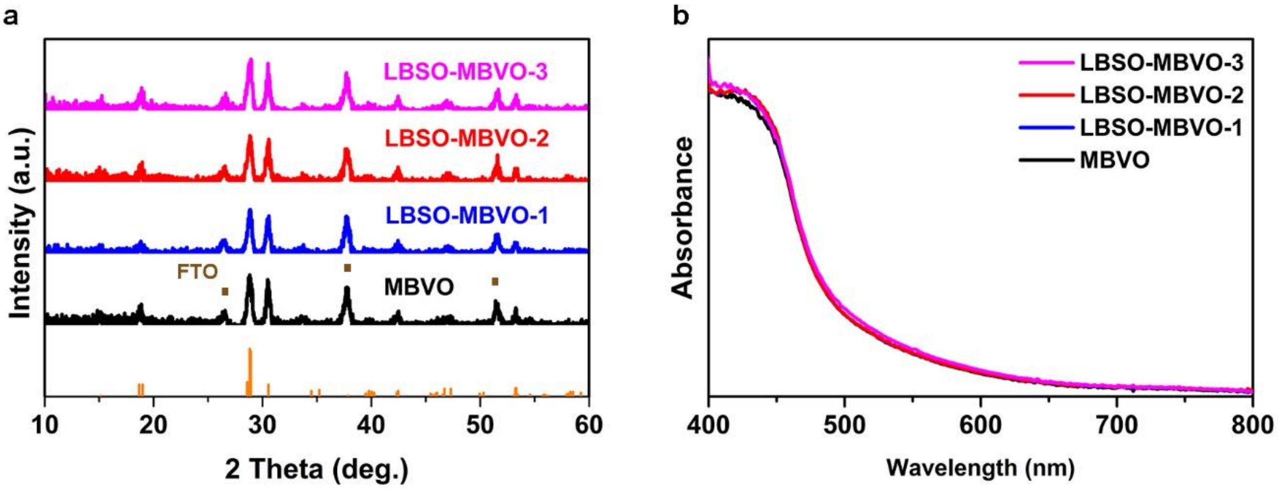

Supplementary Figure 5. Characterization of MBVO and different LBSO-MBVO films. (**a**) Grazing incidence XRD patterns and (**b**) UV/Vis spectra.

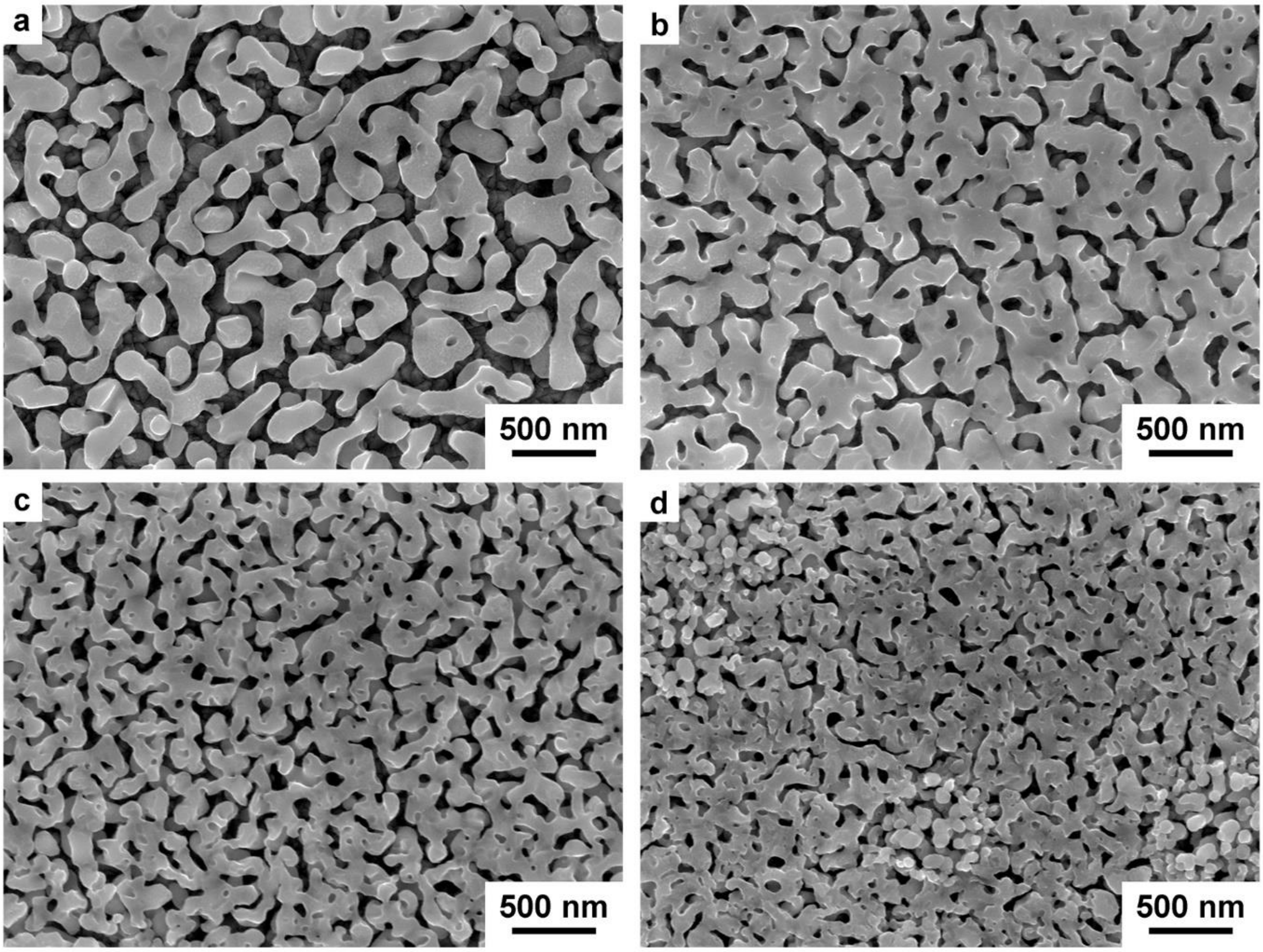

Supplementary Figure 6. SEM images of different LBSO-MBVO films. (**a**) MBVO, (**b**) LBSO-MBVO-1, (**c**) LBSO-MBVO-2 and (**d**) LBSO-MBVO-3.

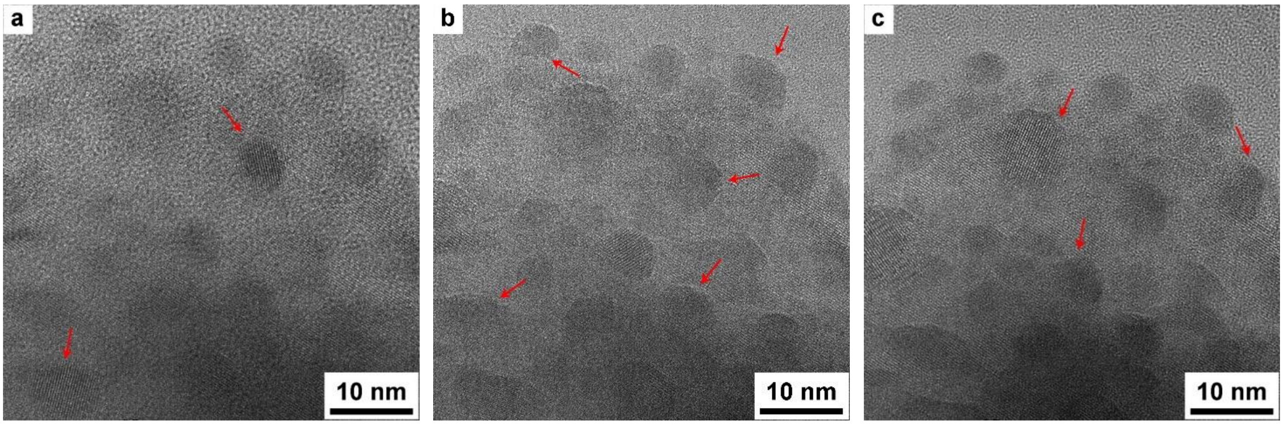

Supplementary Figure 7. Randomly distributed nanocrystals in LBSO-MBVO-2 film with different contrasts/crystal fringes (red arrows) from the BVO matrix. (**a**) Nanocrystals with different crystal fringes, (**b**) nanocrystals with different contrasts and (**c**) nanocrystals with different contrasts and crystal fringes.

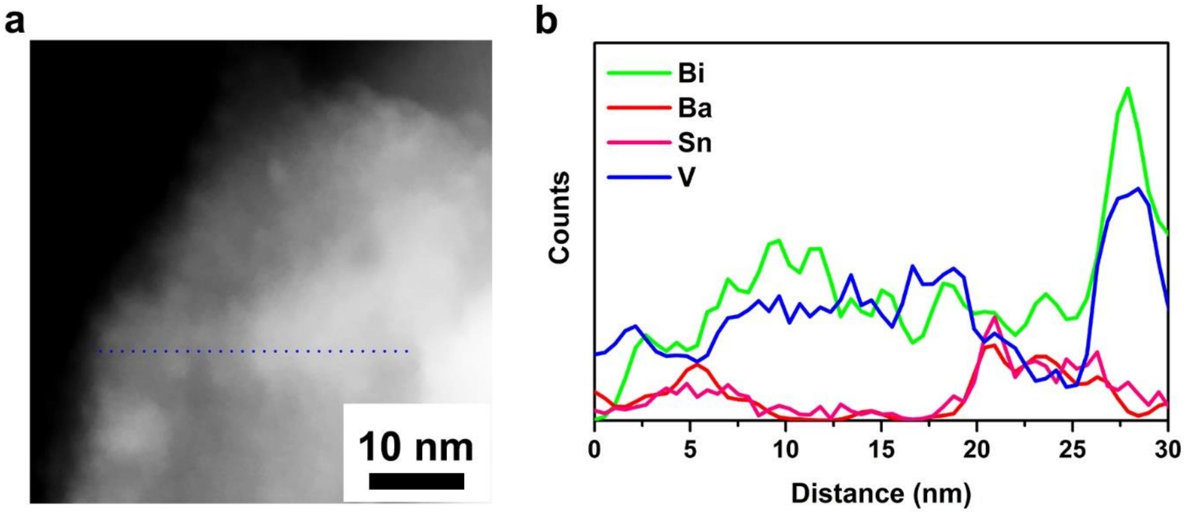

Supplementary Figure 8. Elemental analysis of LBSO-MBVO-2 film. (**a**) HAADF image and (**b**) corresponding EDX-line scanning.

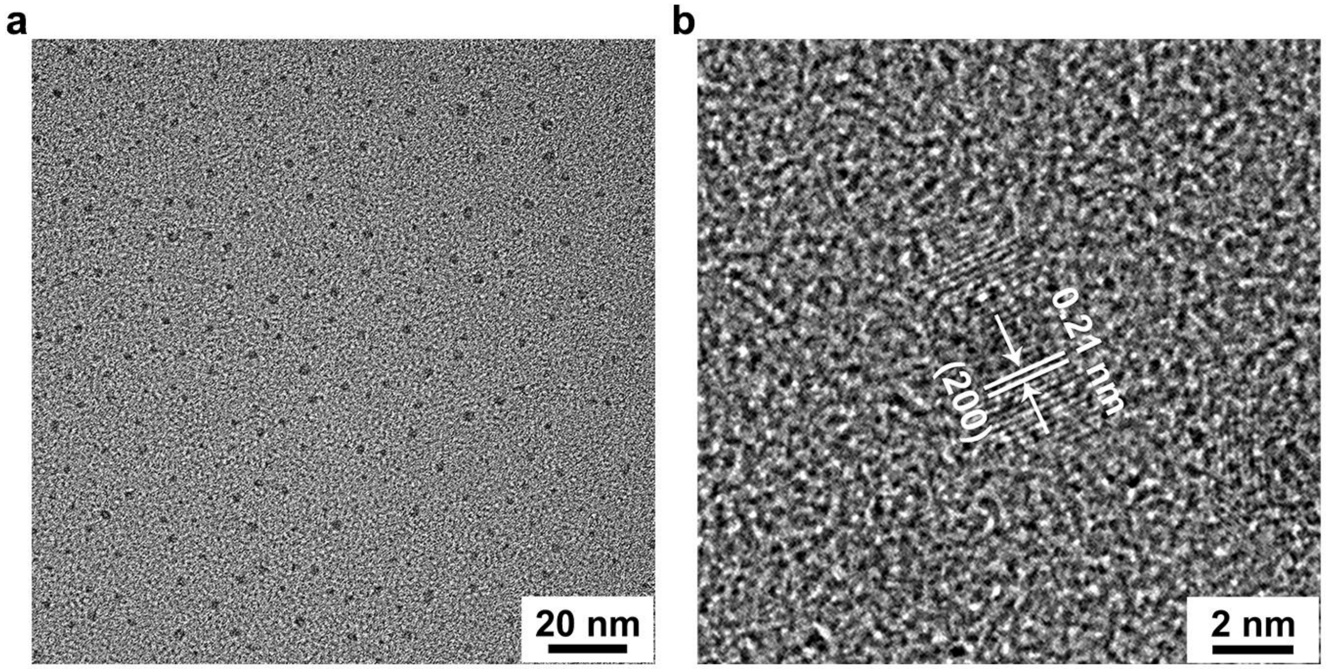

Supplementary Figure 9. A demonstration to show the advantage of LSPC of producing LBSO nanocrystals in desired solvents of ethyl acetate. (**a**) TEM and (**b**) HRTEM image of LBSO nanocrystals.

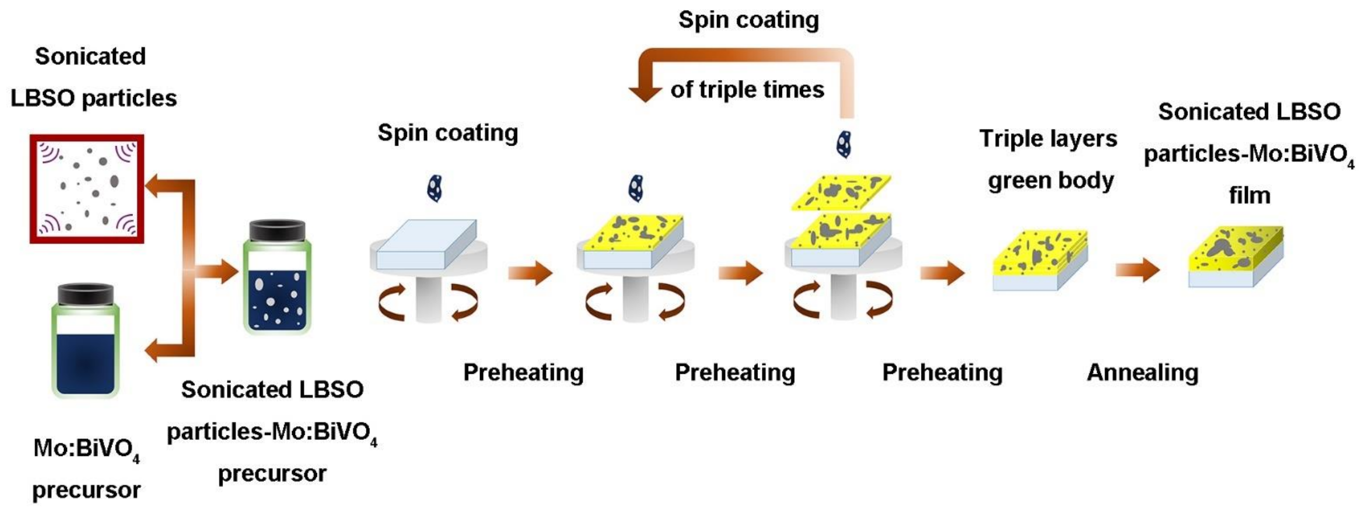

Supplementary Figure 10. Schematic illustration of the fabrication procedure of introducing sonicated LBSO particles in MBVO photoanodes.

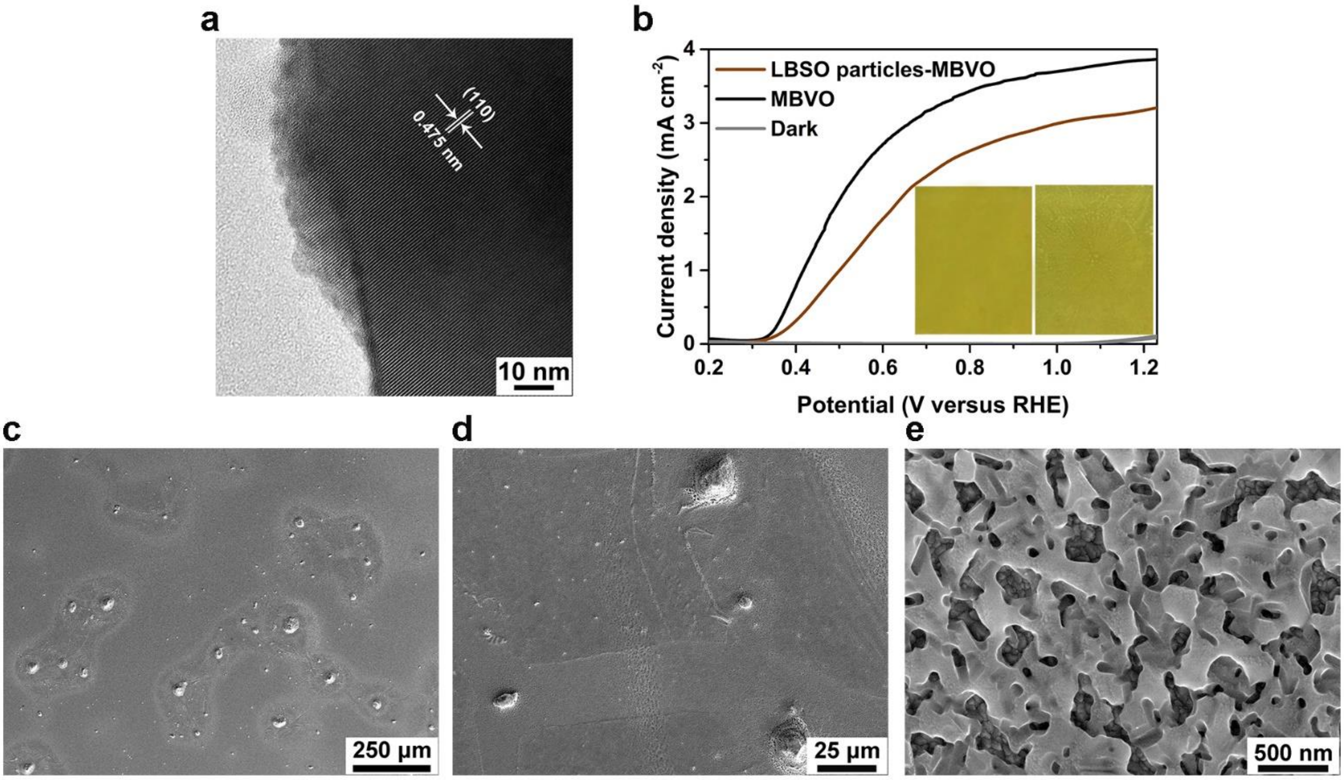

Supplementary Figure 11. Characterization and performance of LBSO particles-MBVO films. (**a**) HRTEM image of MBVO film with LBSO raw nanoparticles introduced in the precursor (without using LSPC), which is denoted as LBSO particles-MBVO film. (**b**) Photocurrent density-potential curves of MBVO films and LBSO particles-MBVO films under AM 1.5G irradiation (insert: photographs of MBVO film (left) and LBSO particles-MBVO film (right)). (**c-e**) SEM images of LBSO particles-MBVO films with different magnifications: (**c**) 250 μm, (**d**) 25 μm and (**e**) 500 nm (around the agglomerations).

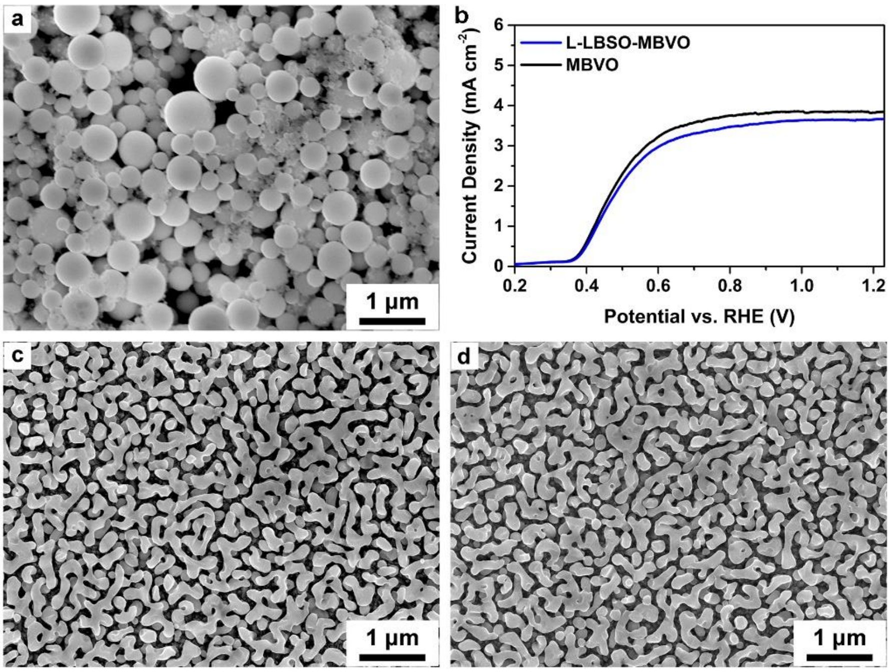

Supplementary Figure 12. Characterization and performance of MBVO and L-LBSO-MBVO films. (**a**) SEM image of the large LBSO particles with the diameter of several hundred nanometers, (**b**) J-V curves of MBVO and L-LBSO-MBVO films, (**c**) SEM image of MBVO film and (**d**) L-LBSO-MBVO film. (L-LBSO represents large LBSO)

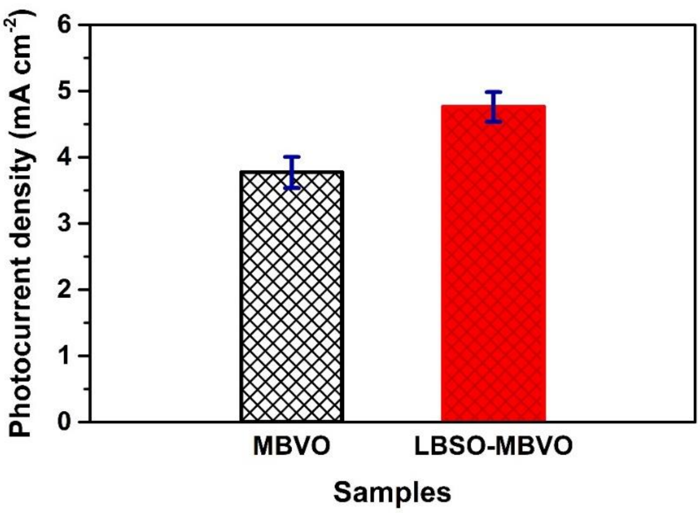

Supplementary Figure 13. Comparation of photocurrent denisities with error bar between MBVO and LBSO-MBVO films.

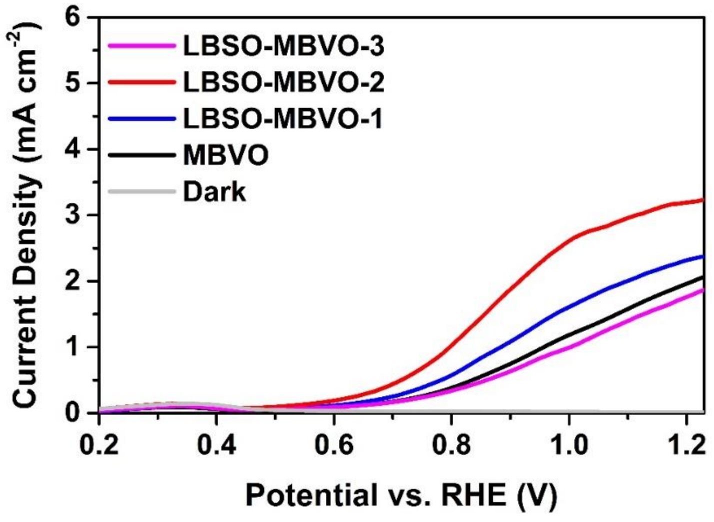

Supplementary Figure 14. J-V curves of different LBSO-MBVO photoanodes under AM 1.5G irradiation in the electrolyte without Na_2SO_3.

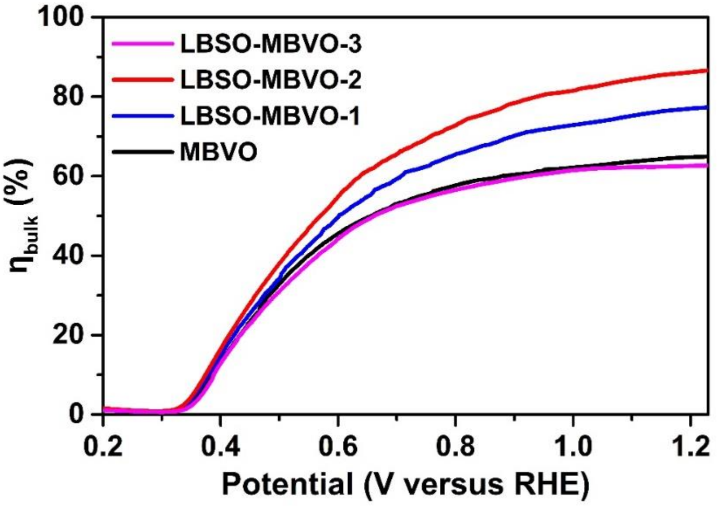

Supplementary Figure 15. Charge transfer efficiencies in the bulk of MBVO and different LBSO-MBVO photoanodes.

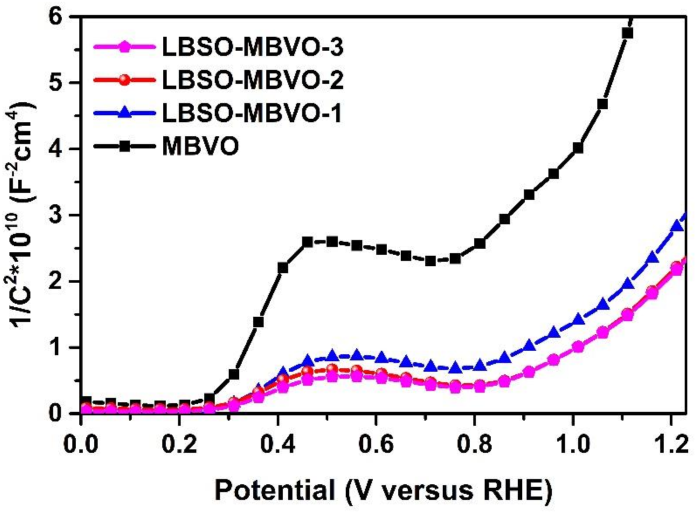

Supplementary Figure 16. MS curves of MBVO and different LBSO-MBVO films.

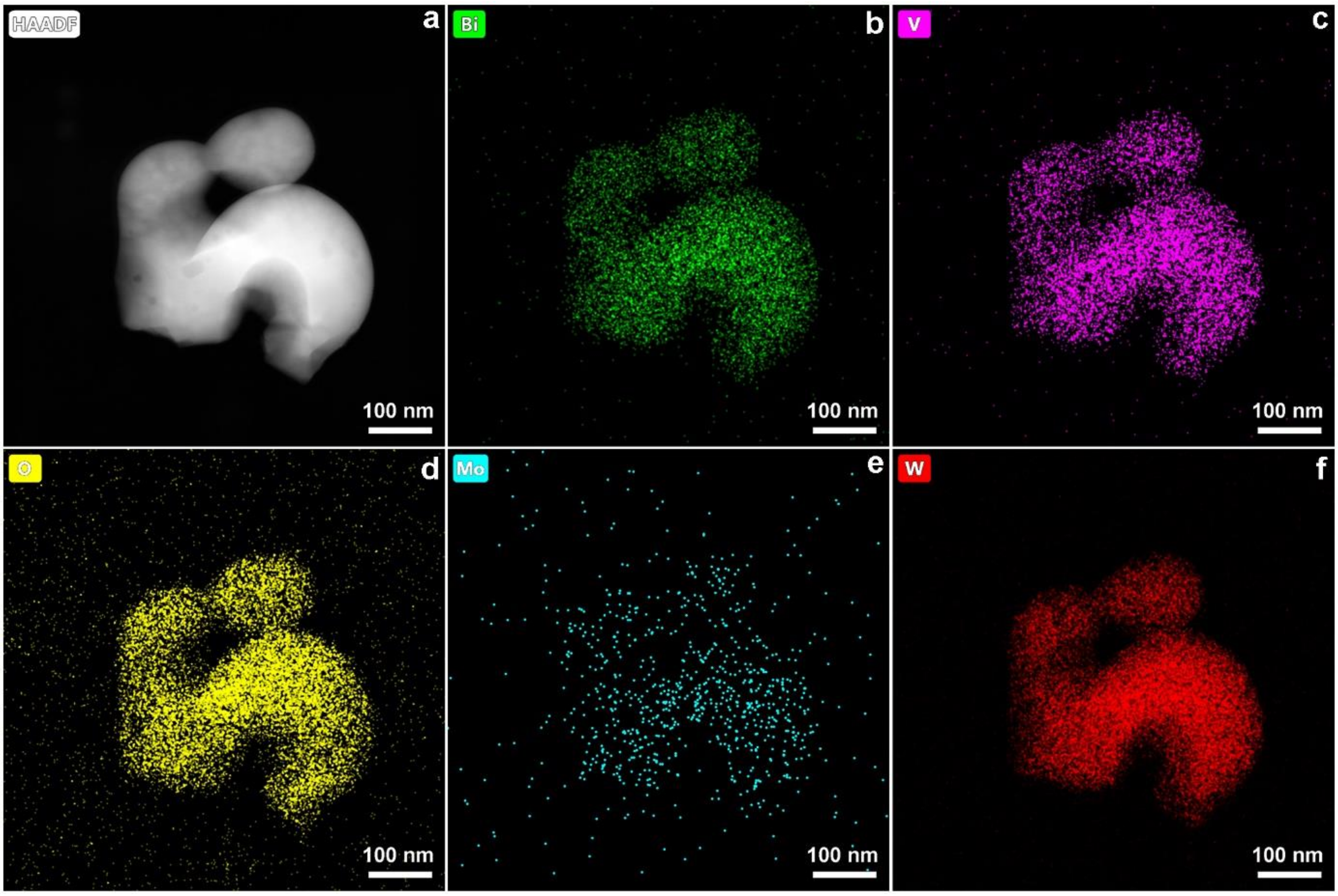

Supplementary Figure 17. TEM-EDS analysis of WO_3-MBVO film. (**a**) HAADF image, (**b**) Bi, (**c**) V, (**d**) O, (**e**) Mo and (**f**) W mapping.

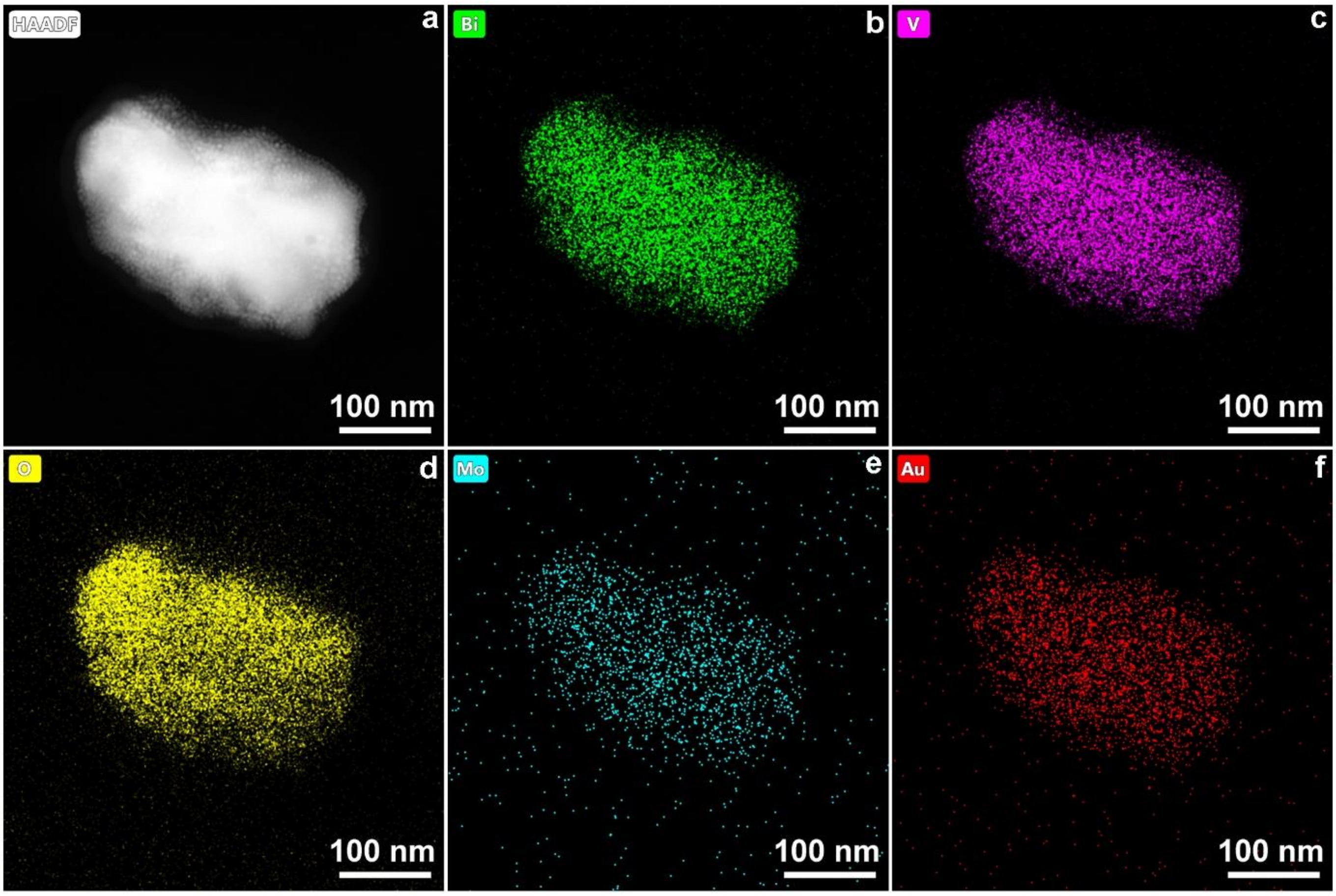

Supplementary Figure 18. TEM-EDS analysis of Au-MBVO film. (**a**) HAADF image, (**b**) Bi, (**c**) V, (**d**) O, (**e**) Mo and (**f**) Au mapping.

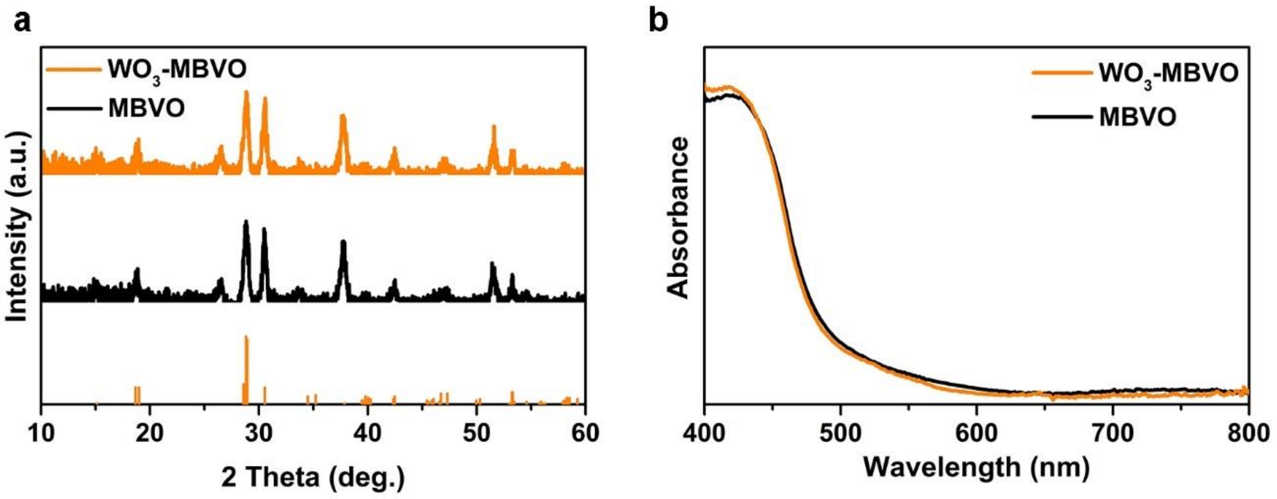

Supplementary Figure 19. Characterization of MBVO and WO_3-MBVO films. (**a**) Grazing incidence XRD patterns and (**b**) UV/Vis spectra.

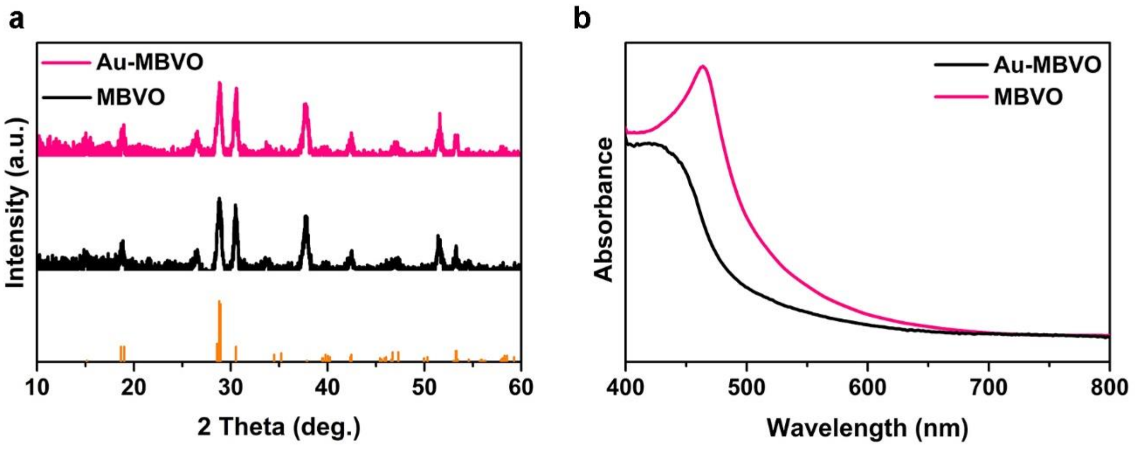

Supplementary Figure 20. Characterization of MBVO and Au-MBVO films. (**a**) Grazing incidence XRD patterns and (**b**) UV/Vis spectra.

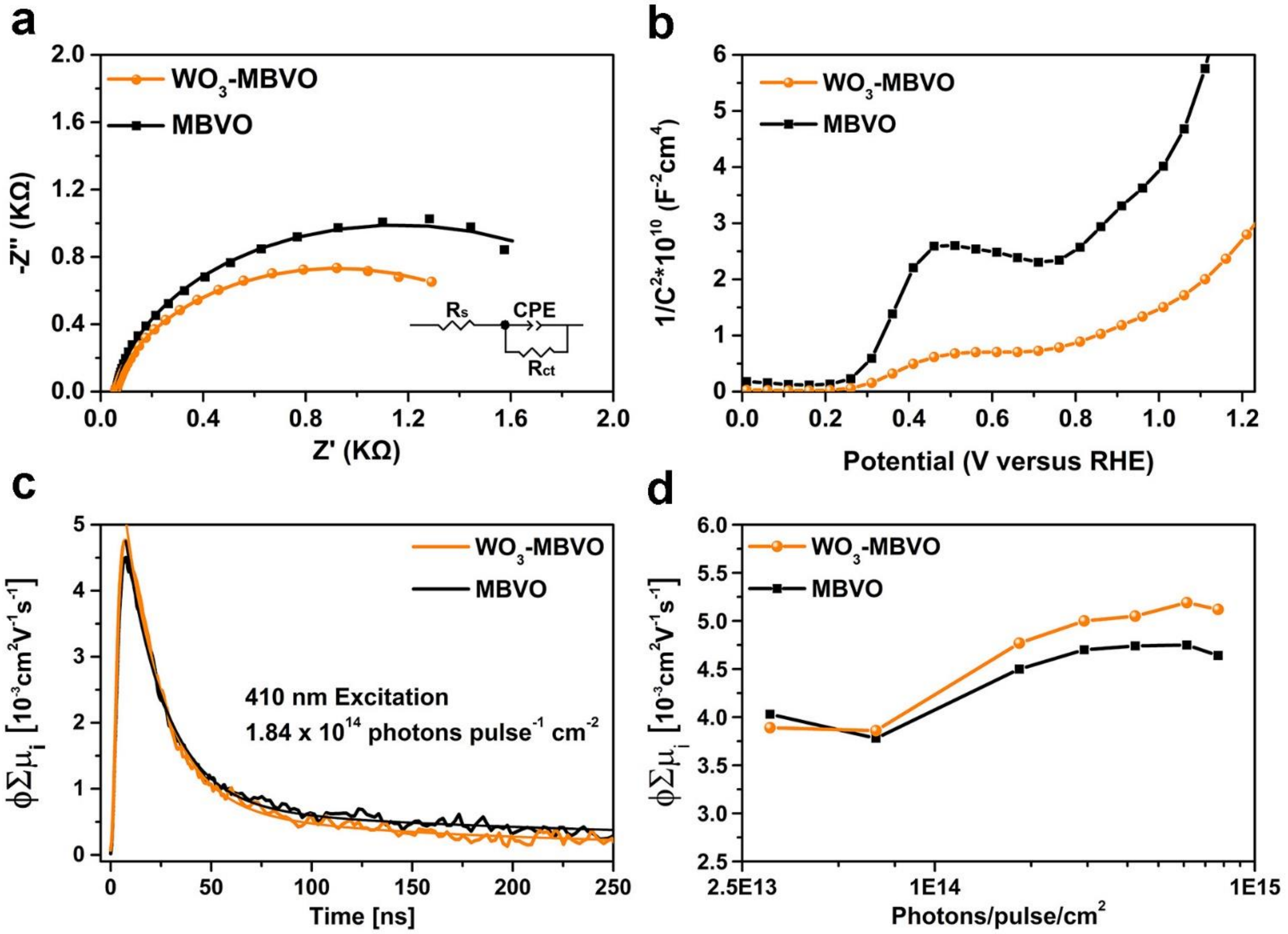

Supplementary Figure 21. Performance of MBVO and WO_3-MBVO films. (**a**) EIS curves, (**b**) MS curves, (**c**) Time-resolved microwave conductance signals recorded using a 410 nm laser pulse with a photon flux of 1.84×10^{14} photons pulse^{-1} cm^{-2} and (**d**) Maximum observed TRMC signals as a function of incident photons per laser pulse.

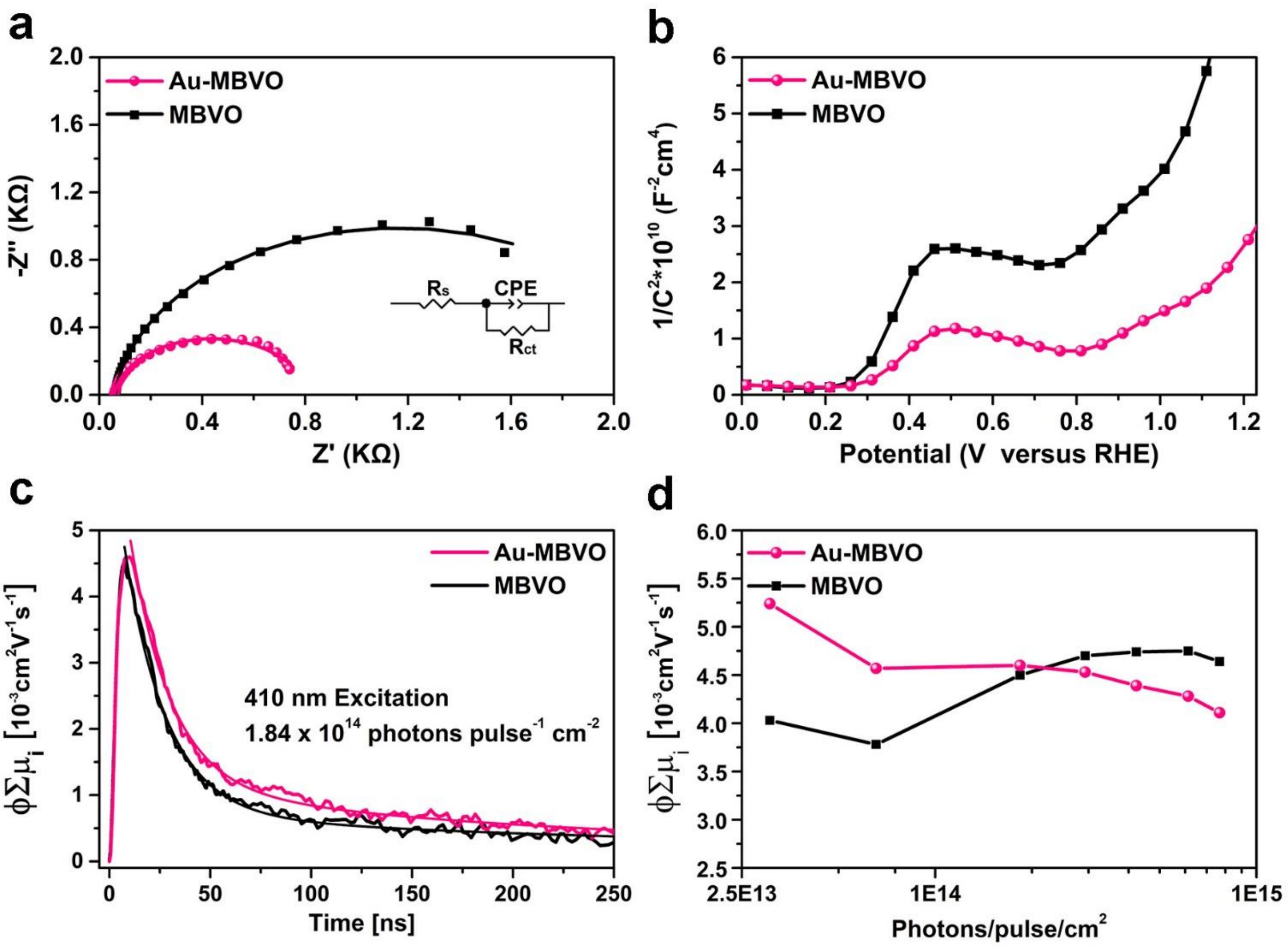

Supplementary Figure 22. Performance of MBVO and Au-MBVO films. (**a**) EIS curves, (**b**) MS curves, (**c**) Time-resolved microwave conductance signals recorded using a 410 nm laser pulse with a photon flux of 1.84×10^{14} photons pulse^{-1} cm^{-2} and (**d**) Maximum observed TRMC signals as a function of incident photons per laser pulse.

Supplementary Table 1. Summary of PEC performance of state-of-the-art $BiVO_4$-based photoanodes.

Photoanode	Electrolyte	J (1.23 V_{RHE})	Ref.
LBSO-MBVO-Double	1.0 M potassium phosphate buffer with 0.1 M Na_2SO_3	6.22 mA cm^{-2}	This work
LBSO-MBVO	1.0 M potassium phosphate buffer with 0.1 M Na_2SO_3	5.15 mA cm^{-2}	This work
E-$BiVO_4$-Double	1 M potassium borate electrolyte with 0.2 M Na_2SO_3	6.22 mA cm^{-2}	1
E-$BiVO_4$	1 M potassium borate electrolyte with 0.2 M Na_2SO_3	5.38 mA cm^{-2}	1
CQDs-$BiVO_4$	0.5 M Na_2SO_3 aqueous solution with KH_2PO_4 buffer solution	5.99 mA cm^{-2}	2
Twin structure-$BiVO_4$	0.1 M $K_2B_4O_7 \cdot 4H_2O$ containing 0.1 M Na_2SO_3	3.2 mA cm^{-2}	3
Multi-layer $BiVO_4$	0.2 M Na_2SO_4 aqueous solution with 2 M Na_2SO_3	5.8 mA cm^{-2}	4
E-$BiVO_4$	1 M potassium borate electrolyte with 0.2 M Na_2SO_3	3.4 mA cm^{-2}	5

Supplementary Table 2. The equivalent circuit fitted results of EIS data in Fig. 3f.

Photoelectrode	R_s	R_{ct}
MBVO	60.55	2199
LBSO-MBVO-1	59.30	1192
LBSO-MBVO-2	57.32	980
LBSO-MBVO-3	58.74	1808

Supplementary Table 3. Carrier mobilities, lifetimes and diffusion lengths of MBVO and LBSO-MBVO-2 films. All values were measured using a 410 nm laser pulse with a photon flux of 1.84×10^{14} photons $pulse^{-1}$ cm^{-2}.

Photoelectrode	Carrier mobility	Carrier lifetime		Diffusion length	
	μ [10^{-3} cm^2 V^{-1} s^{-1}]	τ_1 [ns]	τ_2 [ns]	L_{D1} [nm]	L_{D2} [nm]
MBVO	4.5	21	276	15	56
LBSO-MBVO-2	4.2	20	227	15	48

Supplementary Table 4. Carrier densities (N_d) of MBVO and different LBSO-MBVO films got from MS curves in Fig. 3g.

Photoelectrode	$N_d/10^{18}$ cm^{-3}
MBVO	1.896
LBSO-MBVO-1	6.041
LBSO-MBVO-2	8.117
LBSO-MBVO-3	9.668

Supplementary Table 5. The equivalent circuit fitted results of EIS data in Supplementary Fig. 21a.

Photoelectrode	R_s	R_{ct}
MBVO	60.55	2199
WO_3-MBVO	58.79	1722

Supplementary Table 6. Carrier densities (N_d) of MBVO and WO_3-MBVO films got from MS curves in Supplementary Fig. 21b.

Photoelectrode	$N_d/10^{18}$ cm^{-3}
MBVO	1.896
WO_3-MBVO	6.659

Supplementary Table 7. Carrier mobilities, lifetimes and diffusion lengths of MBVO and WO_3-MBVO films. All values were measured using a 410 nm laser pulse with a photon flux of 1.84×10^{14} photons pulse^{-1} cm^{-2}.

Photoelectrode	Carrier mobility	Carrier lifetime		Diffusion length	
	μ [10^{-3} cm^2 V^{-1} s^{-1}]	τ_1 [ns]	τ_2 [ns]	L_{D1} [nm]	L_{D2} [nm]
MBVO	4.5	21	276	15	56
WO_3-MBVO	4.8	20	160	16	44

Supplementary Table 8. The equivalent circuit fitted results of EIS data in Supplementary Fig. 22a.

Photoelectrode	R_s	R_{ct}
MBVO	60.55	2199
Au-MBVO	57.52	758.2

Supplementary Table 9. Carrier densities (N_d) of MBVO and Au-MBVO films got from MS curves in Supplementary Fig. 22b.

Photoelectrode	$N_d/10^{18}$ cm^{-3}
MBVO	1.896
Au-MBVO	4.403

Supplementary Table 10. Carrier mobilities, lifetimes and diffusion lengths of MBVO and Au-MBVO films. All values were measured using a 410 nm laser pulse with a photon flux of 1.84×10^{14} photons pulse^{-1} cm^{-2}.

Photoelectrode	Carrier mobility	Carrier lifetime		Diffusion length	
	μ [10^{-3} cm^2 V^{-1} s^{-1}]	τ_1 [ns]	τ_2 [ns]	L_{D1} [nm]	L_{D2} [nm]
MBVO	4.5	21	276	15	56
Au-MBVO	4.6	20	194	15	47

Supplementary References

1 Wang, S. et al. New $BiVO_4$ dual photoanodes with enriched oxygen vacancies for efficient solar-driven water splitting. *Adv. Mater.* **30**, 1800486 (2018).

2 Ye, K.-H. et al. Carbon quantum dots as a visible light sensitizer to significantly increase the solar water splitting performance of bismuth vanadate photoanodes. *Energy Environ. Sci.* **10**, 772–779 (2017).

3 Huang, M. et al. Twin structure in $BiVO_4$ photoanodes boosting water oxidation performance through enhanced charge separation and transport. *Adv. Energy Mater.* **8**, 1802198 (2018).

4 Wu, J. et al. Multi-layer monoclinic $BiVO_4$ with oxygen vacancies and V^{4+} species for highly efficient visible-light photoelectrochemical applications. *Appl. Catal. B: Environ.* **221**, 187–195 (2018).

5 Wang, S., Chen, P., Yun, J. H., Hu, Y. & Wang, L. An electrochemically treated $BiVO_4$ photoanode for efficient photoelectrochemical water splitting. *Angew. Chem. Int. Ed.* **56**, 8500–8504 (2017).
